# Supplementary material for: Drivers of microplastic pollution in soil sediments at fish landing centers in Sri Vijaya Puram (Port Blair), South Andaman Island
Source: PeerJ. 2025 Sep 5;13:e19965. doi: 10.7717/peerj.19965 (PMC12422275; doi:10.7717/peerj.19965)
Supplement: Supplemental Information 1 — Detailed data on microplastic pollution at fish landing centers in Sri Vijaya Puram; the relative color and shape compositions of microplastics, respectively; percentage proportions of various Land Use and Land Cover (LULC) categories across sampling sites. A correlation matrix linking microplastic abundance with LULC and population density and FTIR spectra results identifying the polymer types of collected microplastics. [file peerj-13-19965-s001.doc]

**Supplimentary Material 1 :** Relative colour composition (%) of microplastics in samples from different fish landing centres.

| **Region** | **Location** | **Blue** | **Green** | **Red** | **Black** | **Purple** | **White** | **Ash** | **Brown** | **Transparent** |
| --- | --- | --- | --- | --- | --- | --- | --- | --- | --- | --- |
| North | Chatham | 13.5 | 4.9 | 12.4 | 12.2 | 10.1 | 13.7 | 13.2 | 10.9 | 9.1 |
| North | Dignabad | 2 | 14.8 | 9.4 | 10.1 | 19.5 | 12.1 | 12.8 | 13.4 | 6 |
| North | Janglighat | 9.5 | 14.6 | 15.4 | 8.4 | 14.1 | 9.2 | 14.6 | 8.1 | 6.2 |
| South | Wandoor | 1.5 | 27.3 | 15.2 | 13.6 | 0 | 30.3 | 0 | 0 | 12.1 |
| South | Guptapara | 4.8 | 12.5 | 12.5 | 13.5 | 1.9 | 23.1 | 11.5 | 9.6 | 10.6 |
| South | Chidiyatapu | 3.4 | 8.7 | 16.8 | 13.5 | 14.4 | 14.9 | 8.2 | 10.6 | 9.6 |

**Supplimentary Material 2 :** Relative shape composition (%) of microplastics in samples from

different fish landing centres.

| **Region** | **Location** | **Film** | **Fragments** | **Fiber** | **Foam** | **Paint Particles** |
| --- | --- | --- | --- | --- | --- | --- |
| North | Chatham | 23.6 | 26.4 | 15.5 | 19.2 | 15.3 |
| North | Dignabad | 9.4 | 26.2 | 12.8 | 28.2 | 23.5 |
| North | Janglighat | 27 | 18.4 | 20.3 | 18.9 | 15.4 |
| South | Wandoor | 37.9 | 16.7 | 28.8 | 6.1 | 10.6 |
| South | Guptapara | 0 | 33.7 | 45.2 | 4.8 | 16.3 |
| South | Chidiyatapu | 18.3 | 49.5 | 28.4 | 0 | 3.8 |

**Supplimentary Material 3 :** Table displaying the percentage proportions of Land Use and Land Cover (LULC) categories for each site

| **Region** | **Site** | **Tree cover** | **Grassland** | **Cropland** | **Built-up** | **Bare / sparse vegetation** | **Water bodies** | **Mangrove** |
| --- | --- | --- | --- | --- | --- | --- | --- | --- |
| North | Chatham | 48.24 | 0.52 | 0.22 | 44.51 | 0.44 | 6.07 | 0 |
| North | Dignabad | 32.85 | 3.77 | 0.03 | 58.73 | 0.13 | 4.49 | 0 |
| North | Janglighat | 47.25 | 1.96 | 0.1 | 47.03 | 1.24 | 2.28 | 0.14 |
| South | Wandoor | 72.97 | 7.16 | 1.2 | 0.24 | 0.45 | 15.49 | 2.48 |
| South | Guptapara | 74.86 | 8.27 | 1.11 | 0.05 | 0.02 | 0.08 | 15.61 |
| South | Chidiyatapu | 75 | 6.7 | 0.48 | 0.1 | 0.03 | 11.66 | 6.04 |

**Supplimentary Material 4 :** The correlation matrix showing the relationship between microplastic abundance, LULC categories, and population density variables within the 1 km buffers.


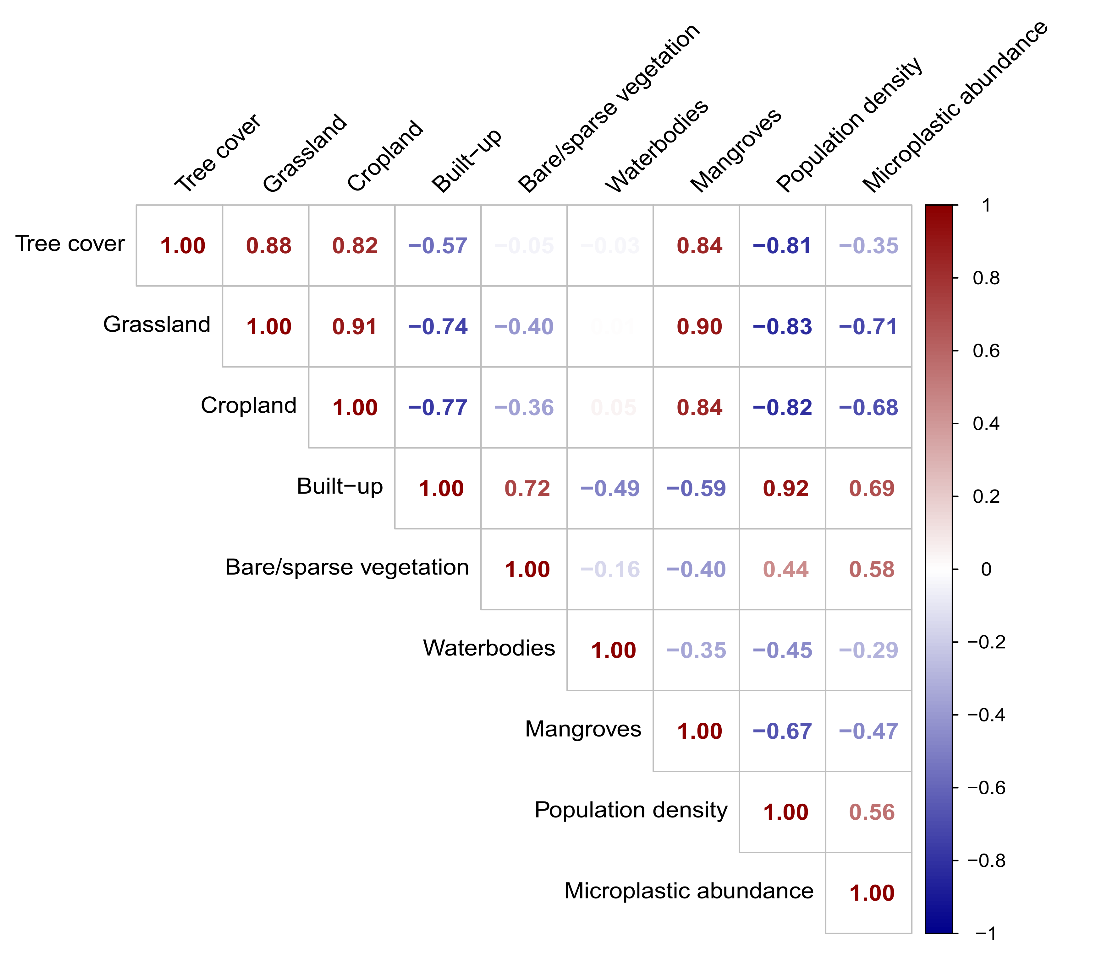


**Supplimentary Material 5:** FTIR spectra of different microplastic polymers.

| **Location: Junglighat** | | | |
| --- | --- | --- | --- |
| **Sl. No** | **Sample ID and Information** | **Type of Microplastic** | **FTIR Result** |
| **1** | Junglighat 1 | ARAMID fiber | 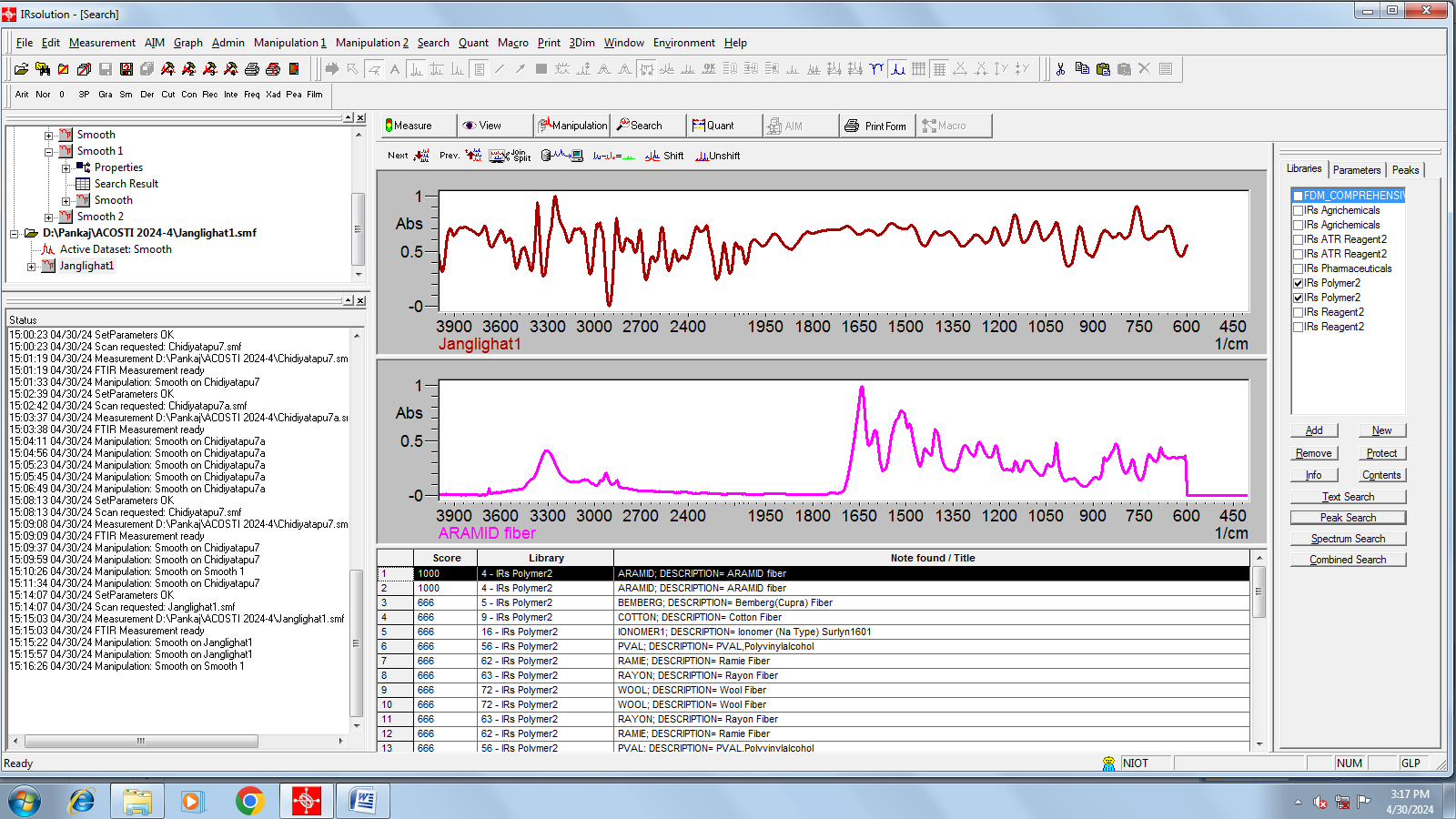 |
| **2** | Junglighat 2 | ARAMID fiber | 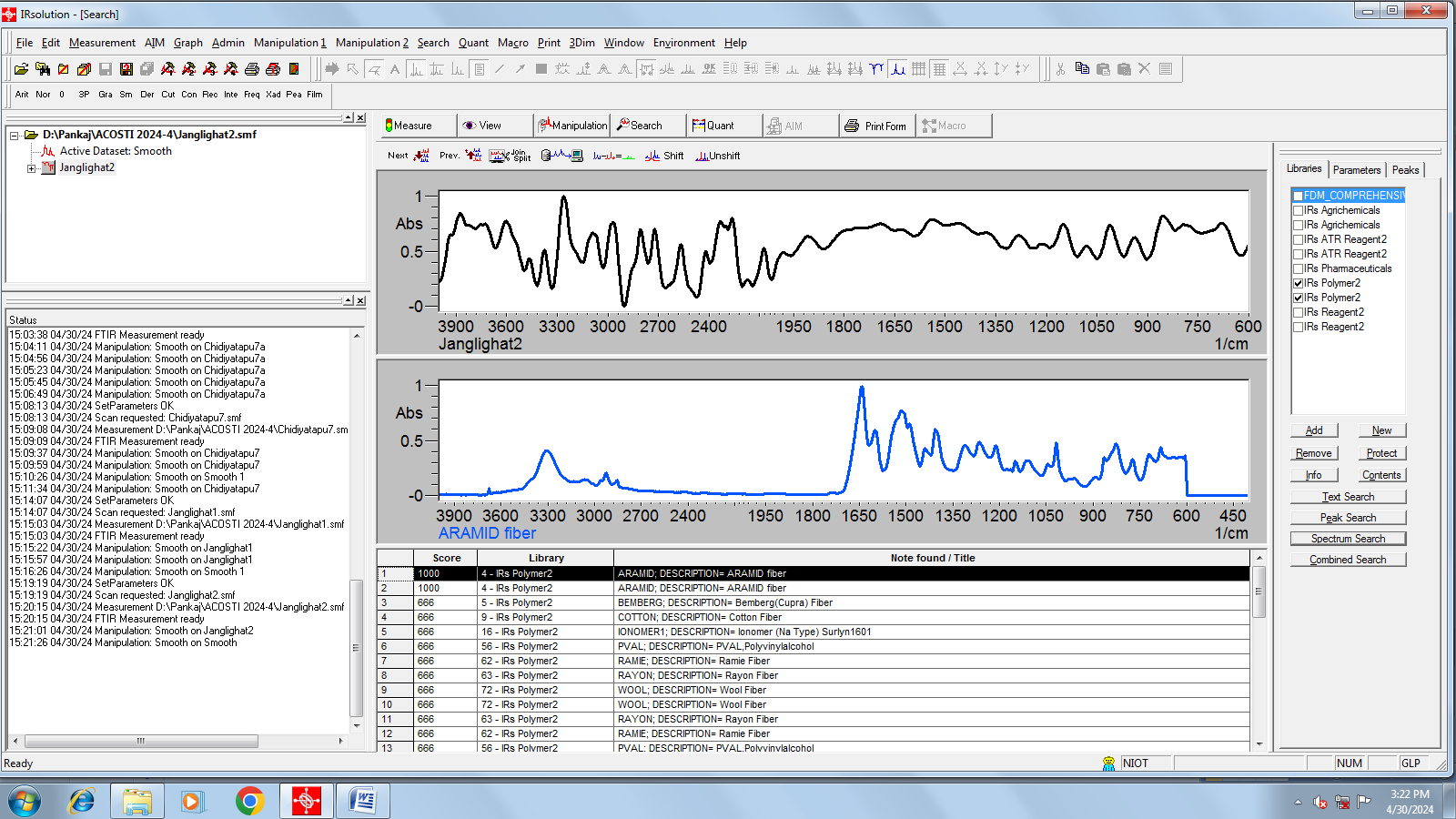 |
| **3** | Junglighat 3 | ARAMID fiber | 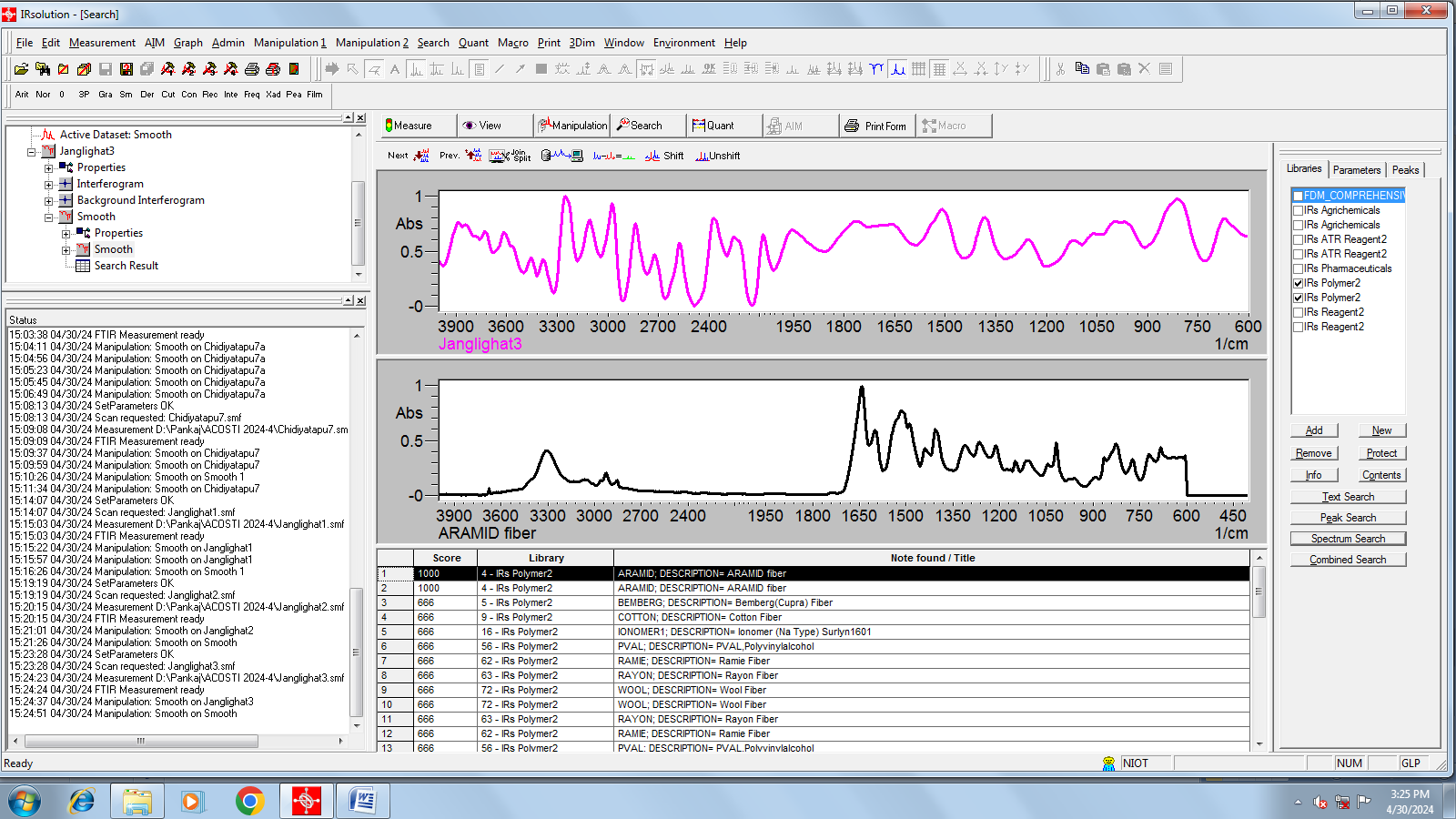 |
| **4** | Junglighat 4 | Acryl fiber | 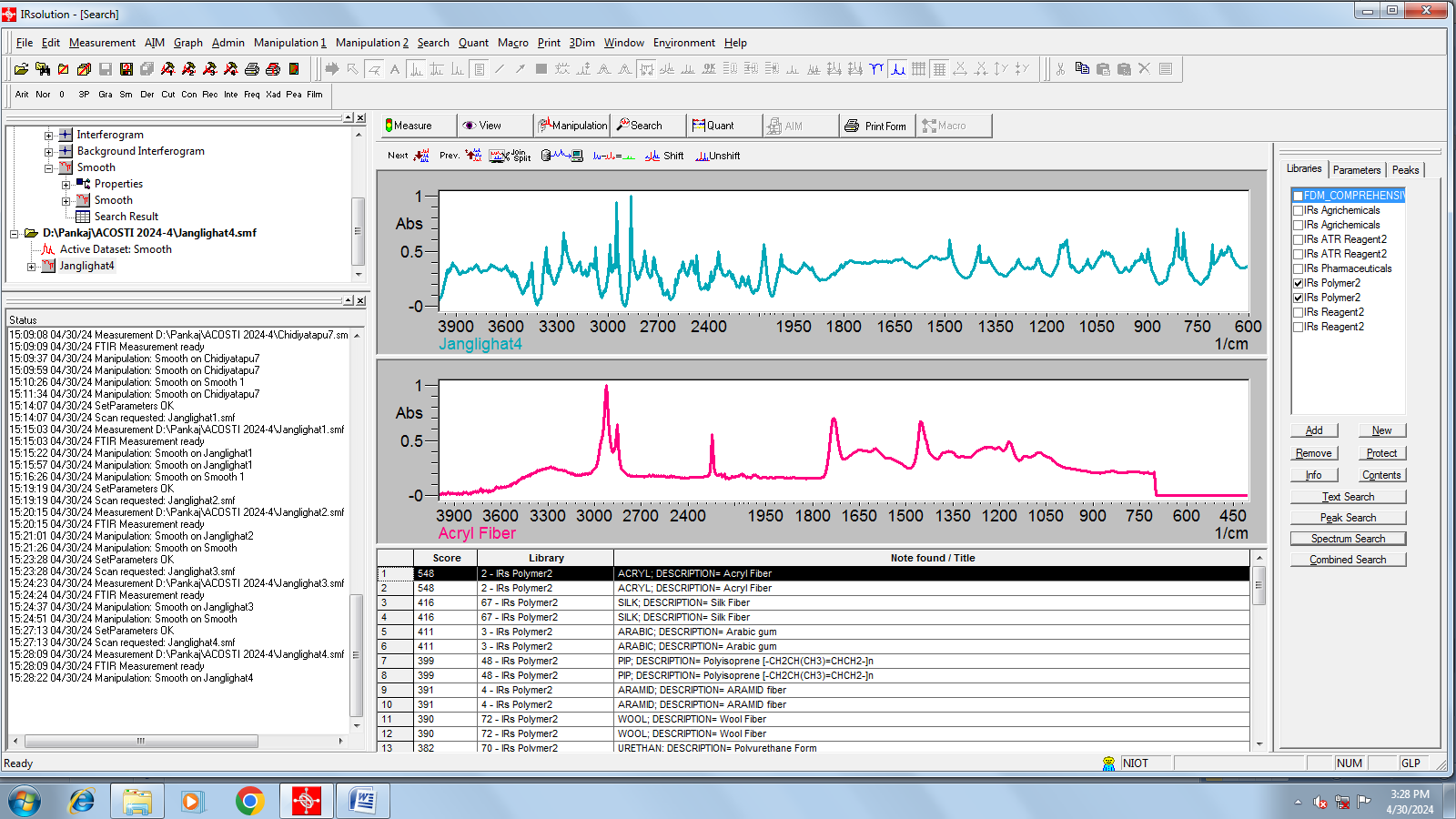 |
| **5** | Junglighat 5 | PVDC, Poly (vinylidene chloride / wrap film) | 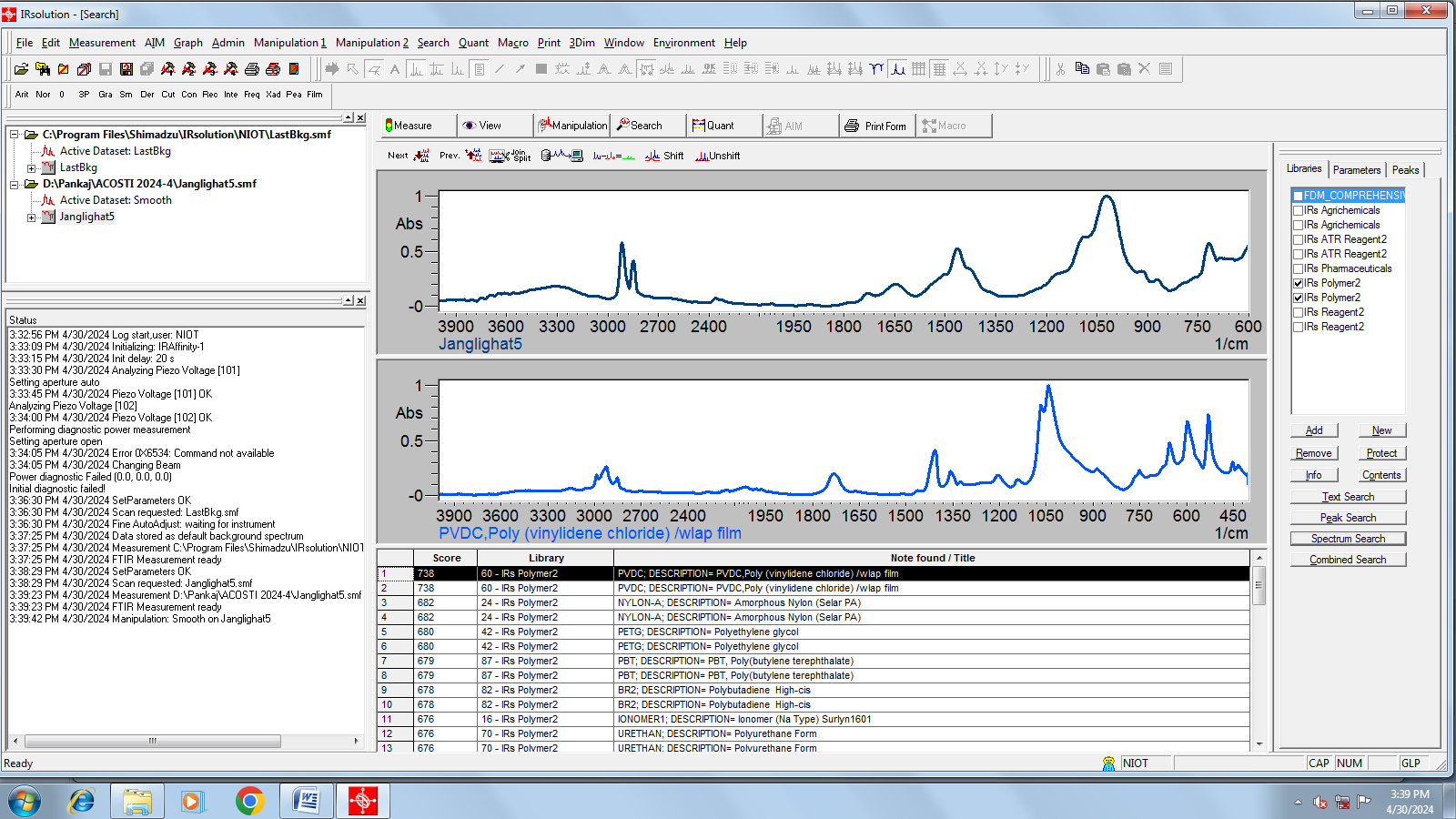 |

| **Location: Chatham** | | | |
| --- | --- | --- | --- |
| **Sl. No** | **Sample ID and Information** | **Type of Microplastic** | **FTIR Result** |
| **1** | Chatham 1 | Acrylonitrile – butadiene Rubber | 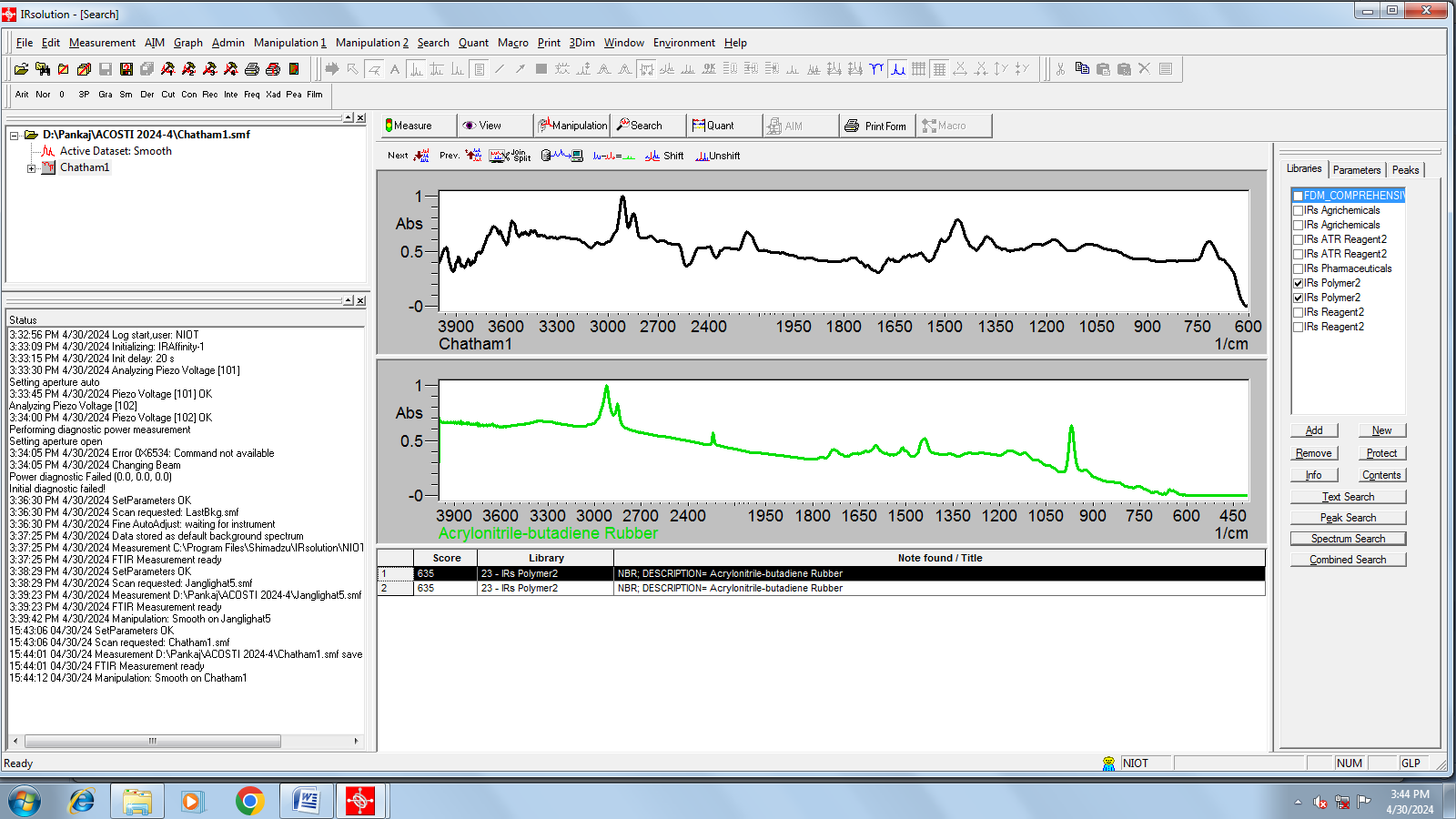 |
| **2** | Chatham 2 | ARAMID fiber | 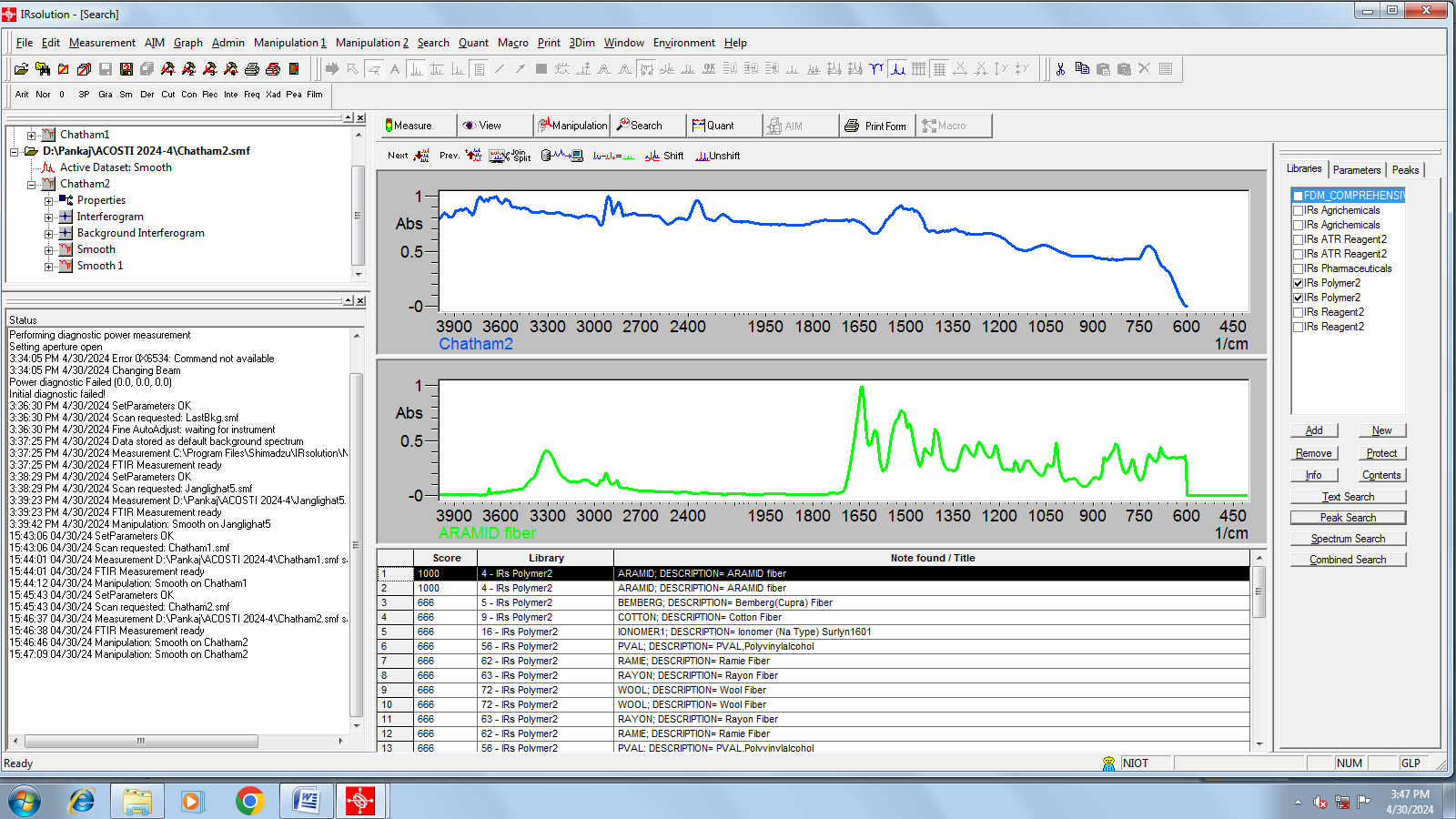 |
| **3** | Chatham 3 | Cellulose | 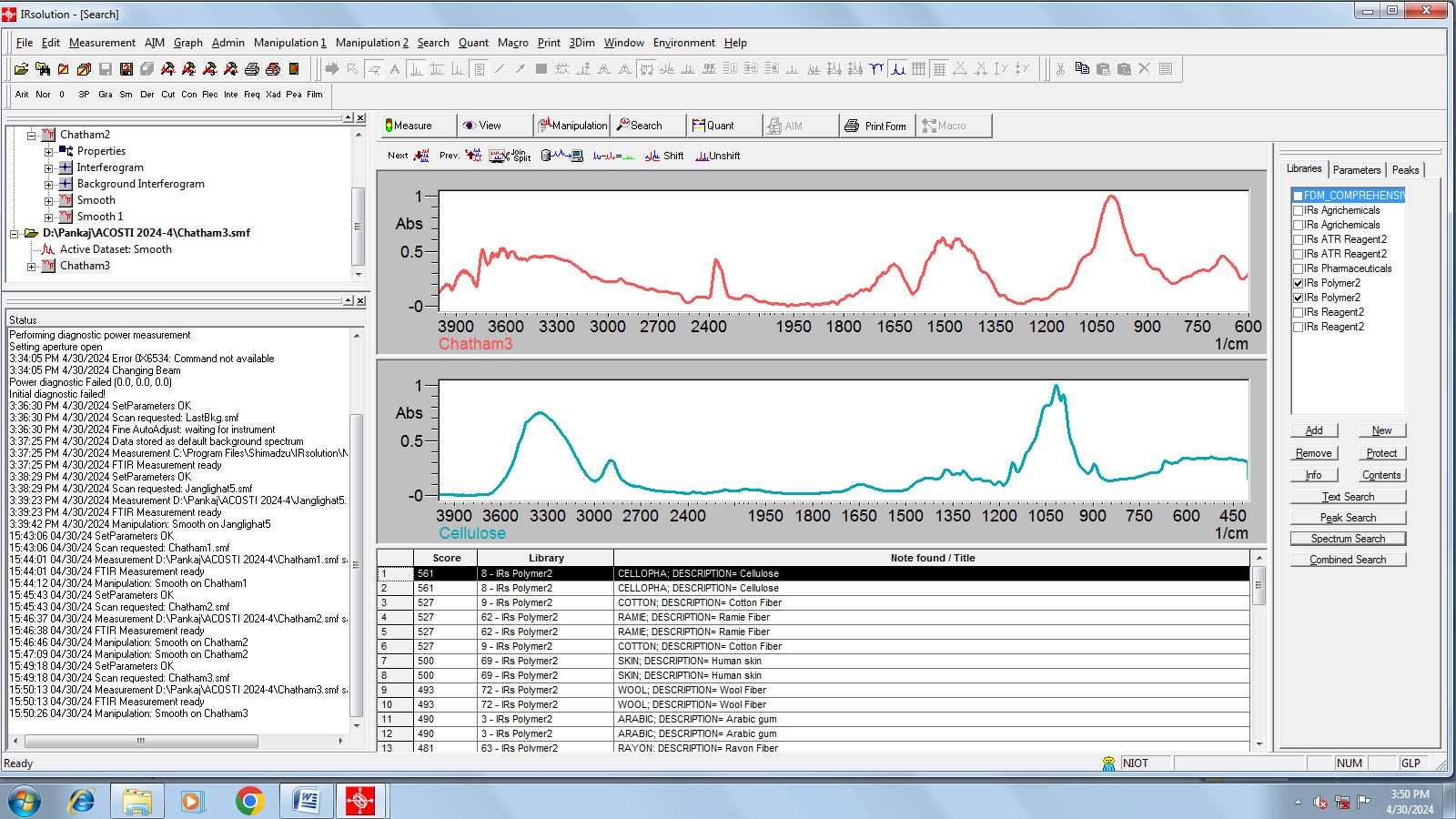 |
| **4** | Chatham 4 | Wool Fiber | 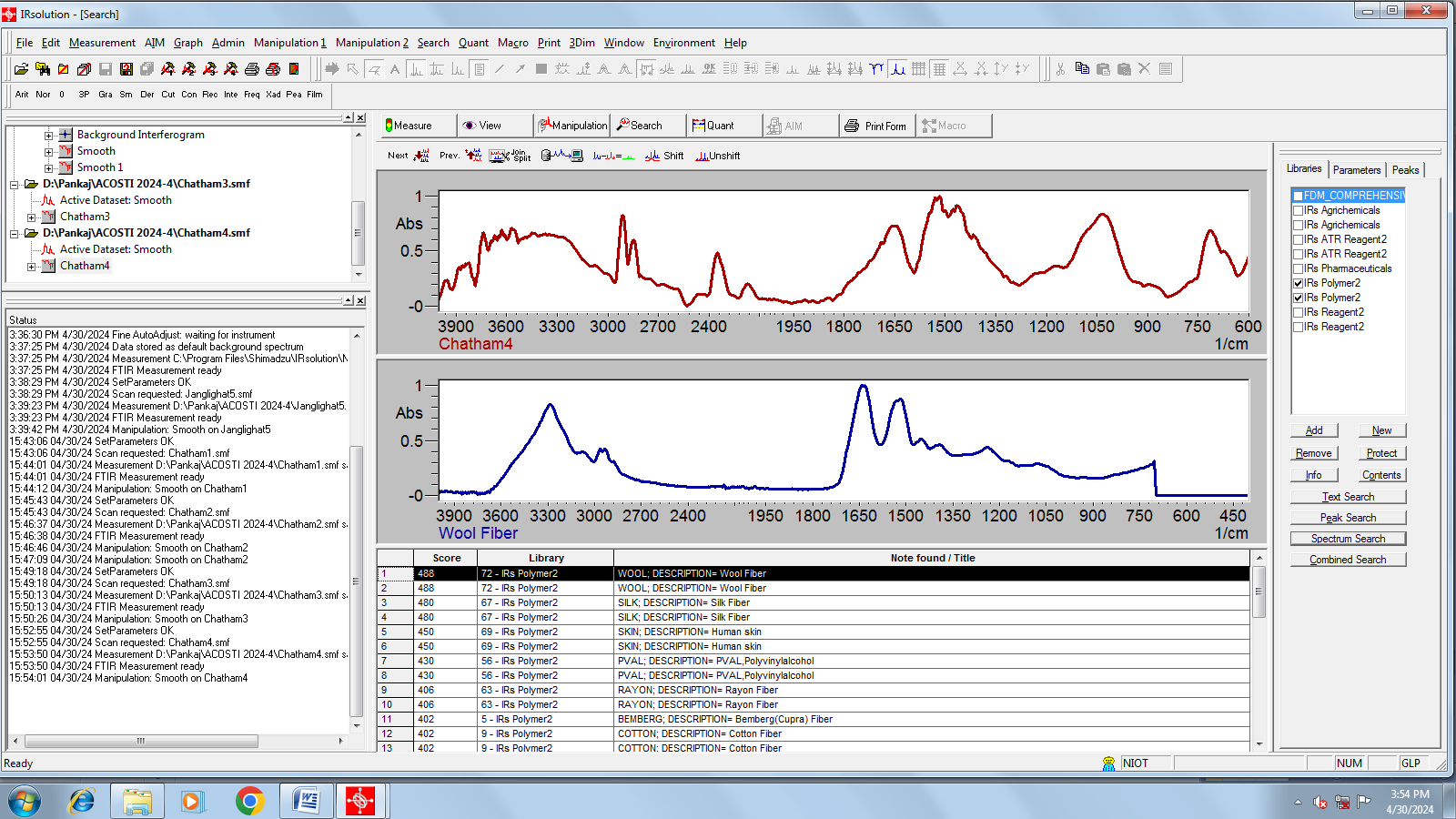 |

| **Location: Wandoor** | | | |
| --- | --- | --- | --- |
| **Sl. No** | **Sample ID and Information** | **Type of Microplastic** | **FTIR Result** |
| **1** | Wandoor 1 | ARAMID fiber | 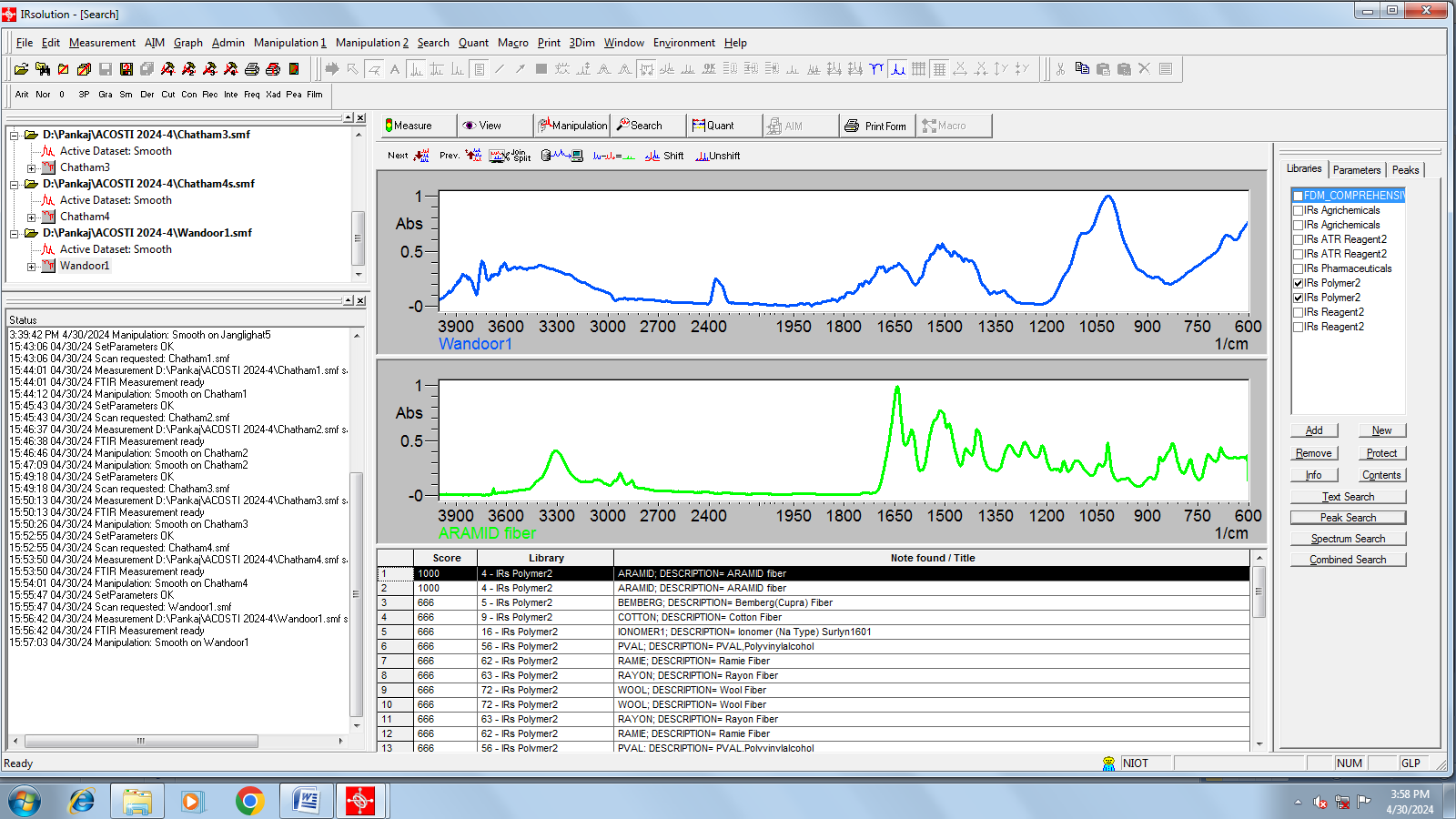 |
| **2** | Wandoor 2 | Acrylonitrile – butadiene Rubber | 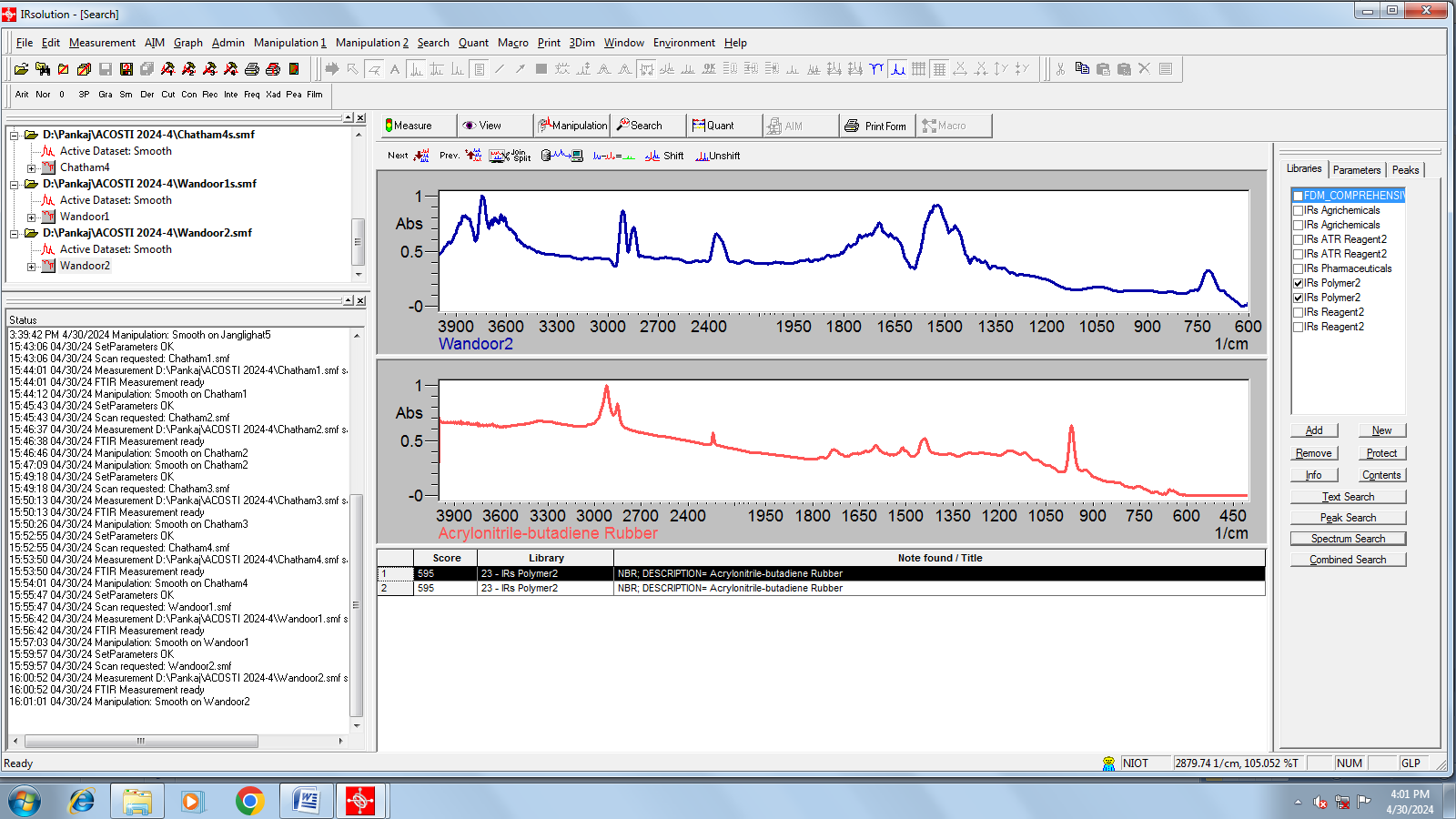 |
| **3** | Wandoor 3 | ARAMID fiber | 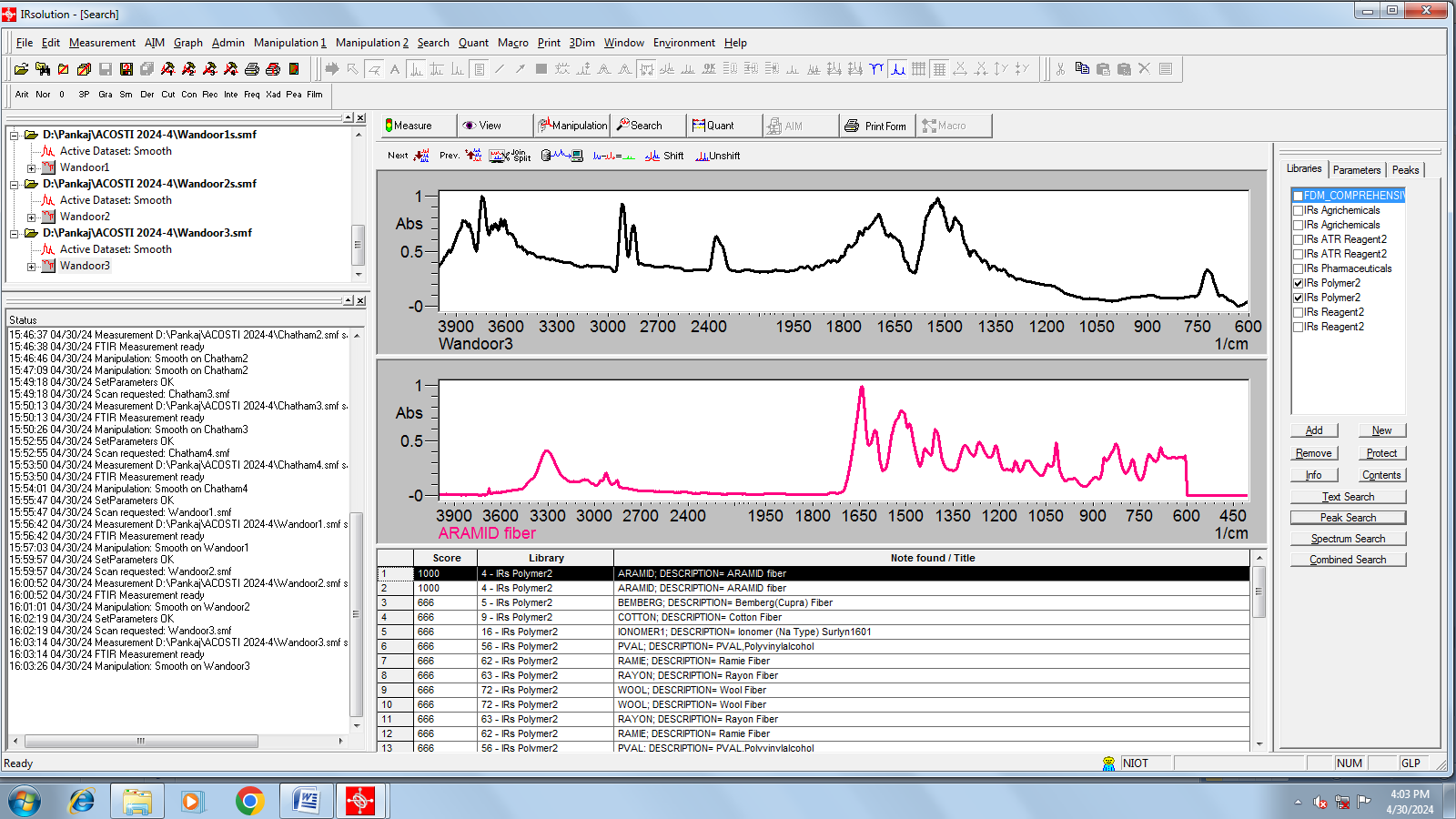 |
| **4** | Wandoor 4 | Polyisoprene [-CH2CH(CH3) =CHCH2-] n | 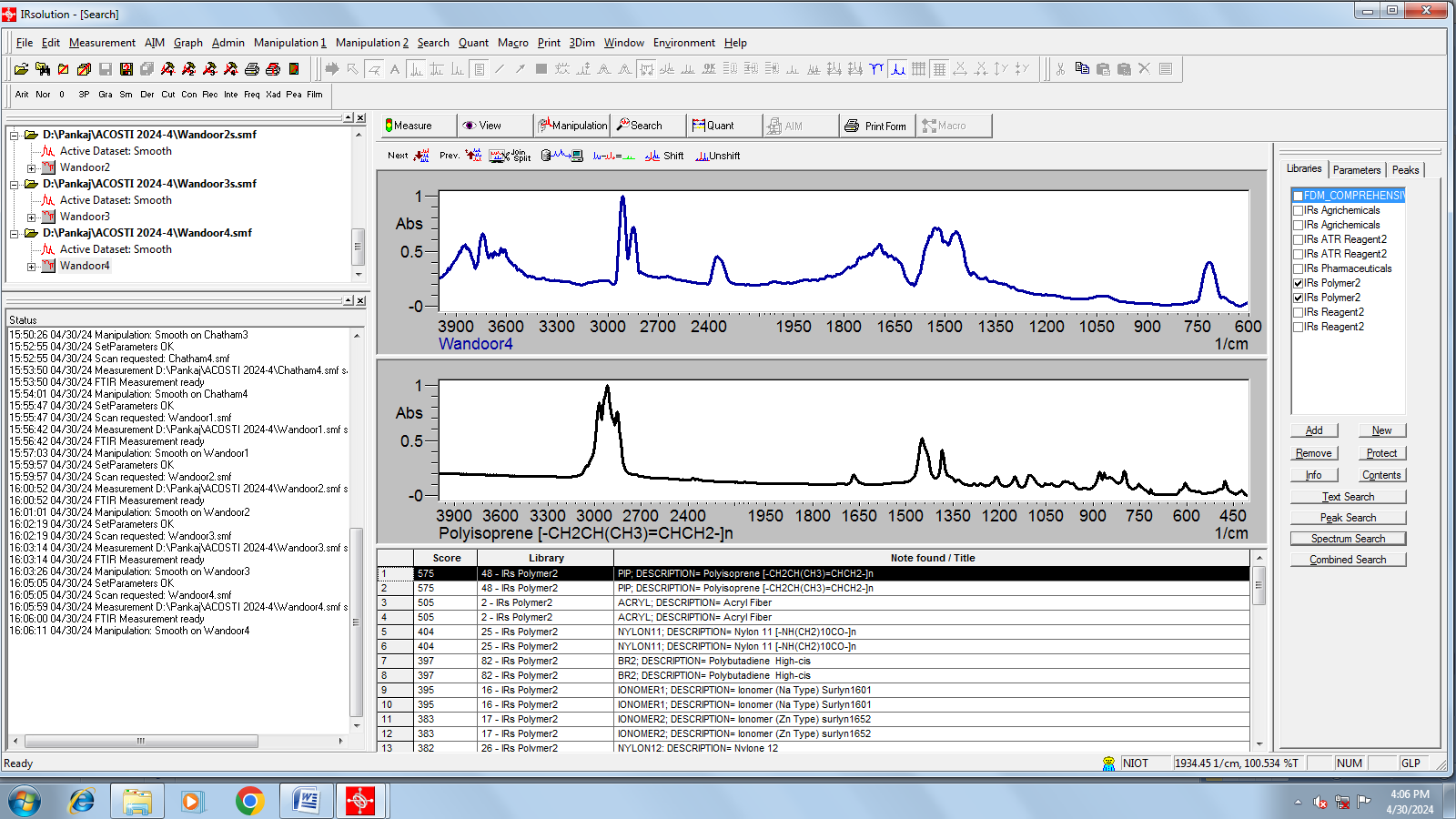 |
| **5** | Wandoor 5 | Polyisoprene [-CH2CH(CH3) =CHCH2-] n | 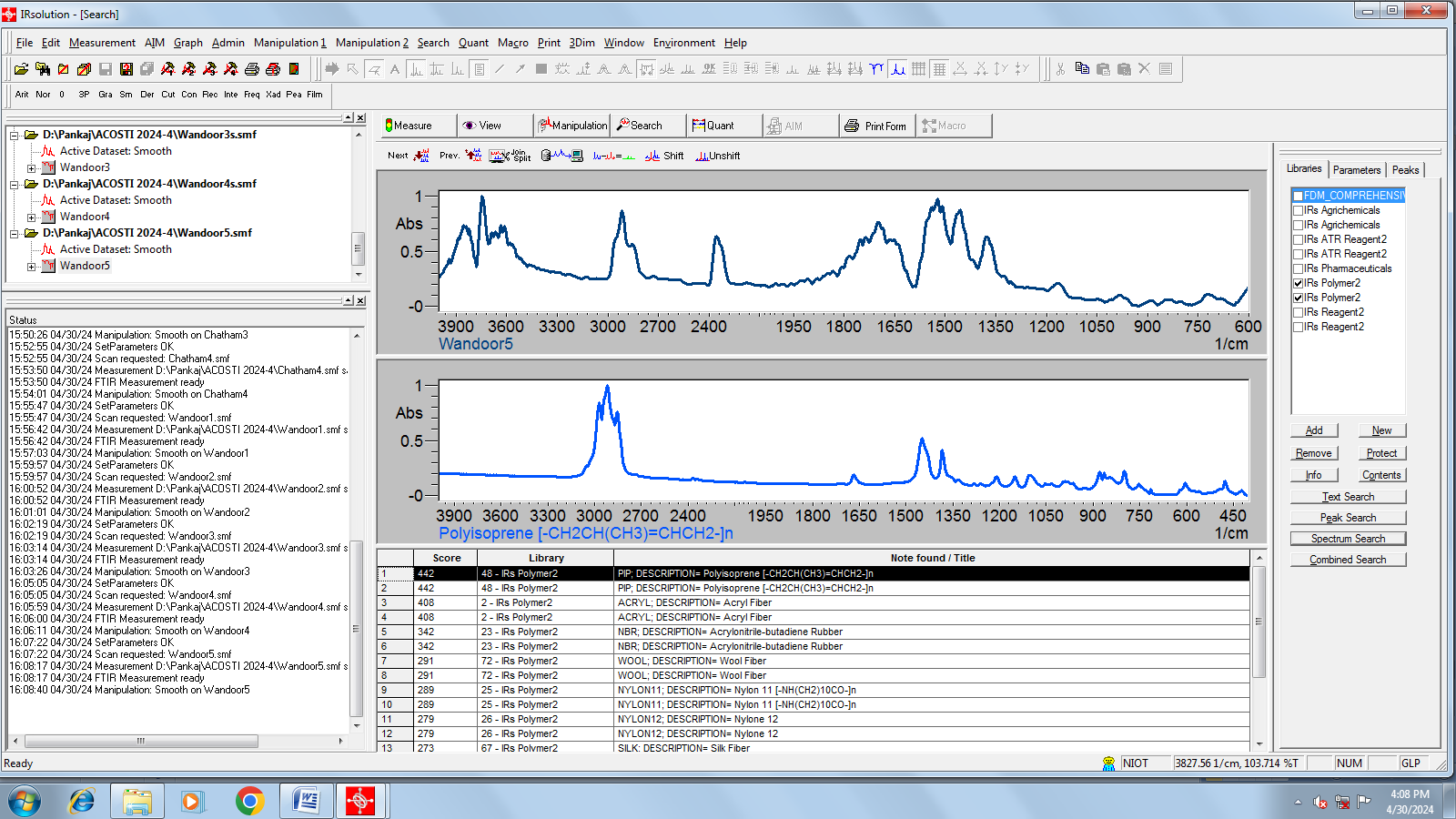 |

| **Location: Guptapara** | | | |
| --- | --- | --- | --- |
| **Sl. No** | **Sample ID and Information** | **Type of Microplastic** | **FTIR Result** |
| **1** | Guptapara 1 | ARAMID fiber | 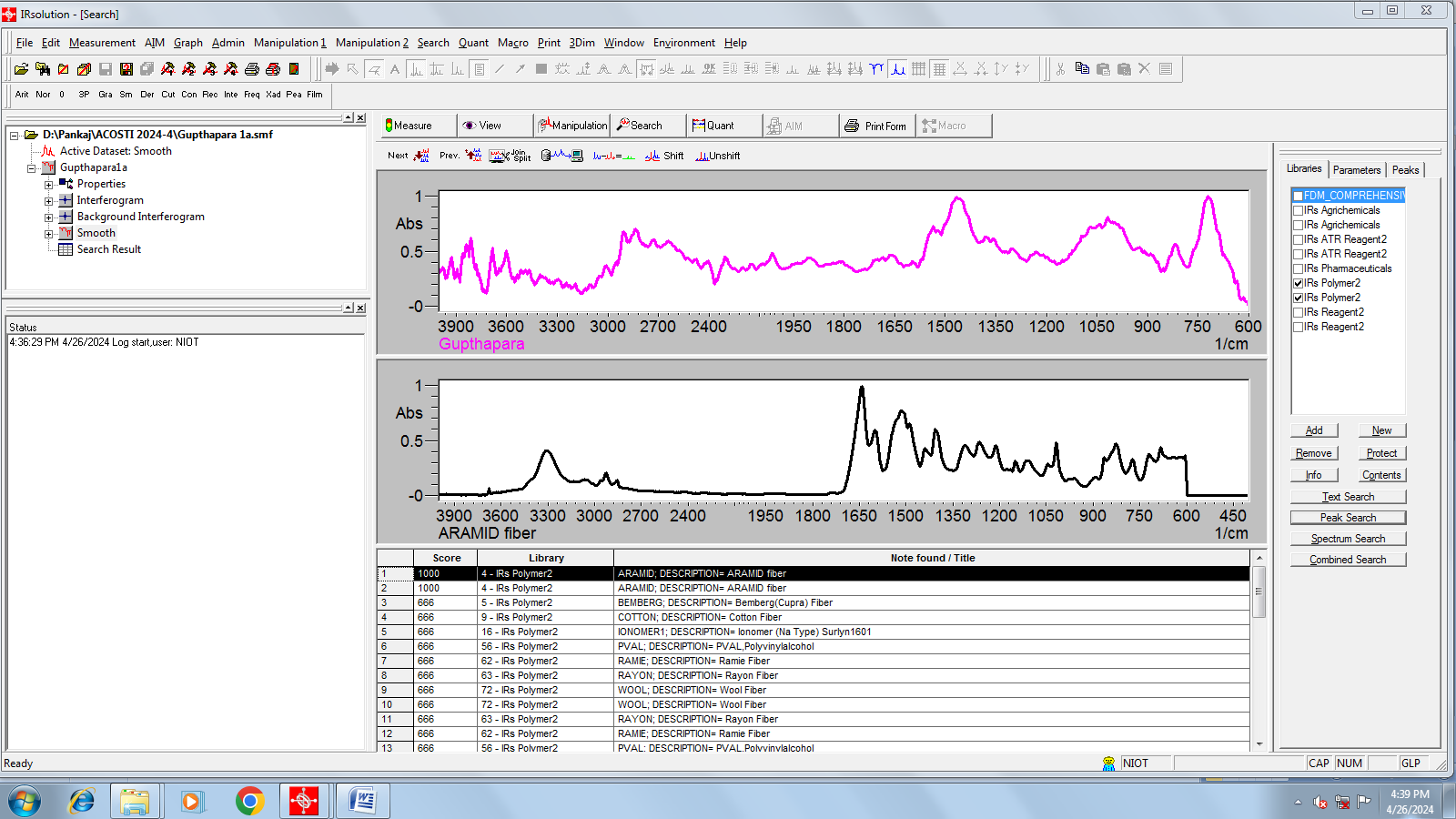 |
| **2** | Guptapara 2 | Arabic gum | 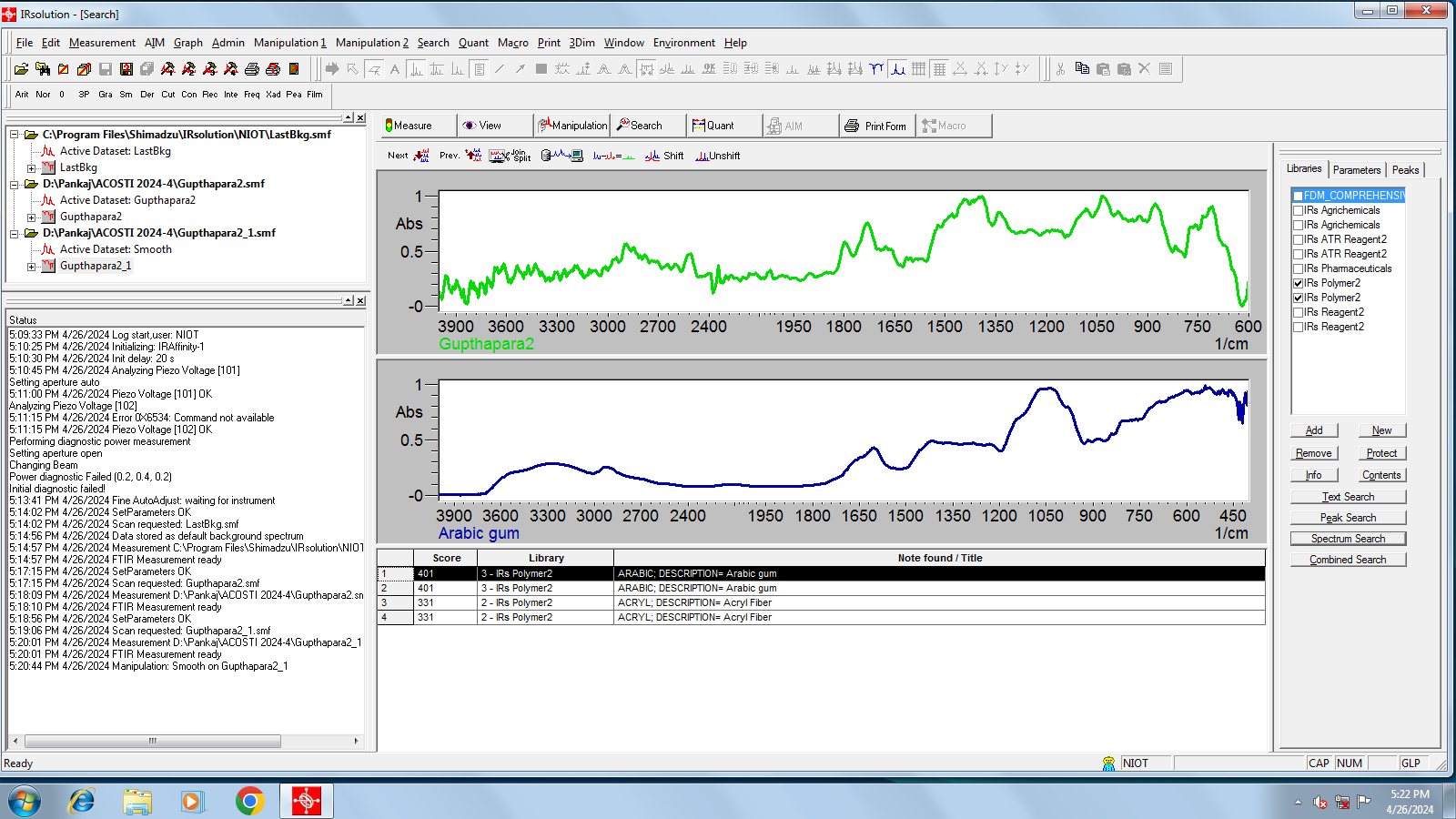 |
| **3** | Guptapara 3 | ARAMID fiber | 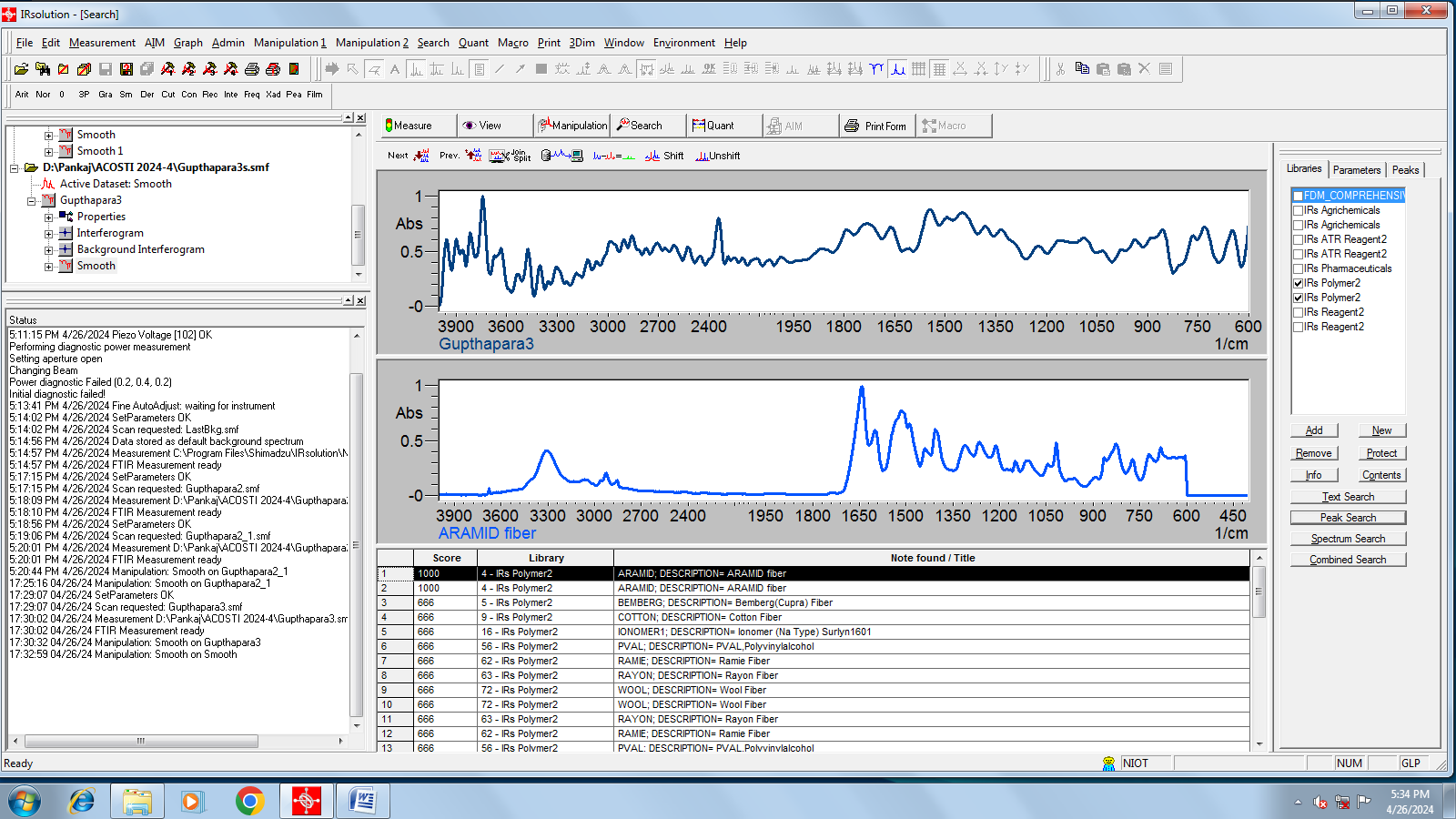 |

| **Location: Chidiyatapu** | | | |
| --- | --- | --- | --- |
| **Sl. No** | **Sample ID and Information** | **Type of Microplastic** | **FTIR Result** |
| **1** | Chidiyatapu 1aa | Acryl fiber | 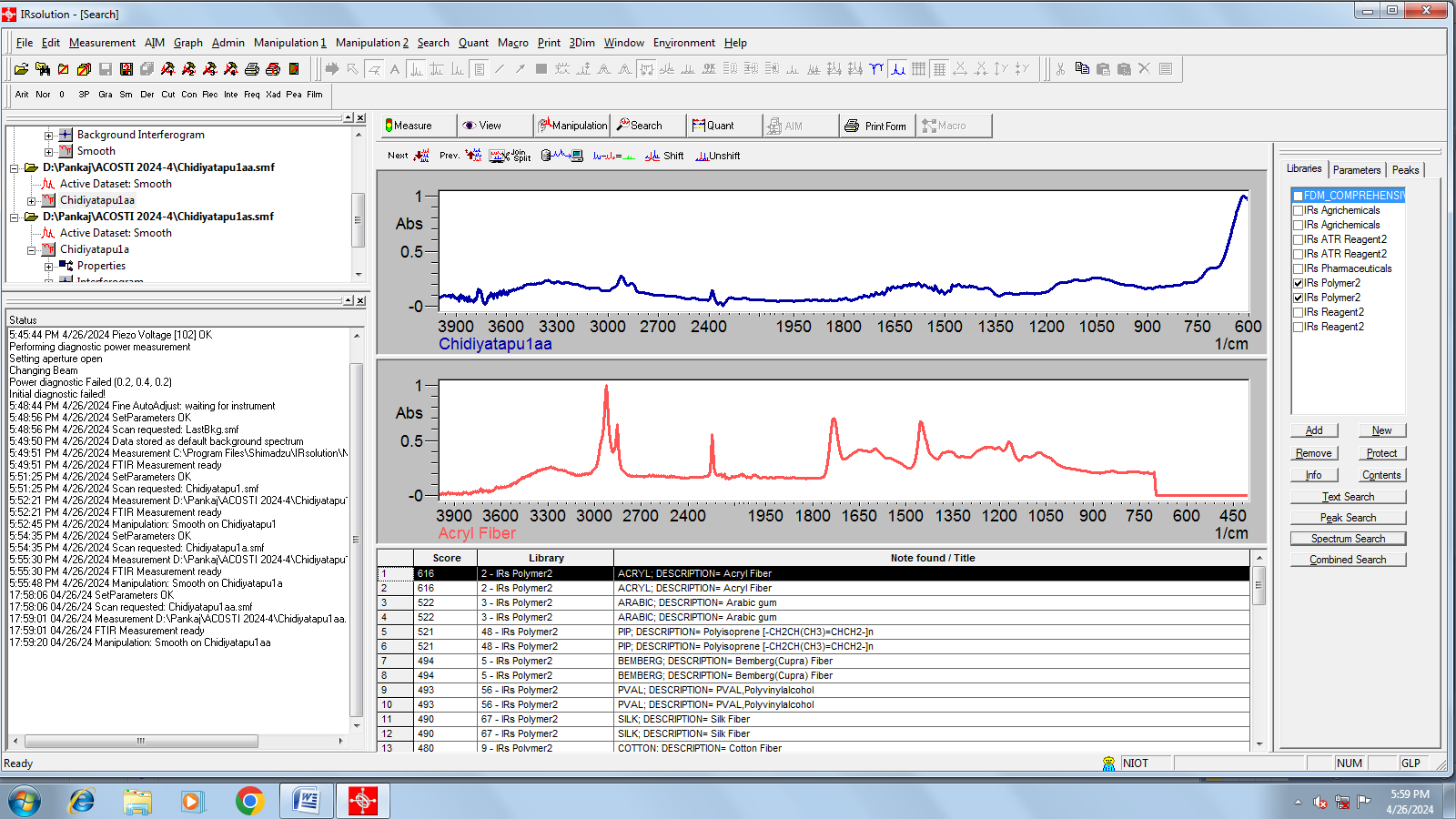 |
| **2** | Chidiyatapu 2 | Polyethylene glycol | 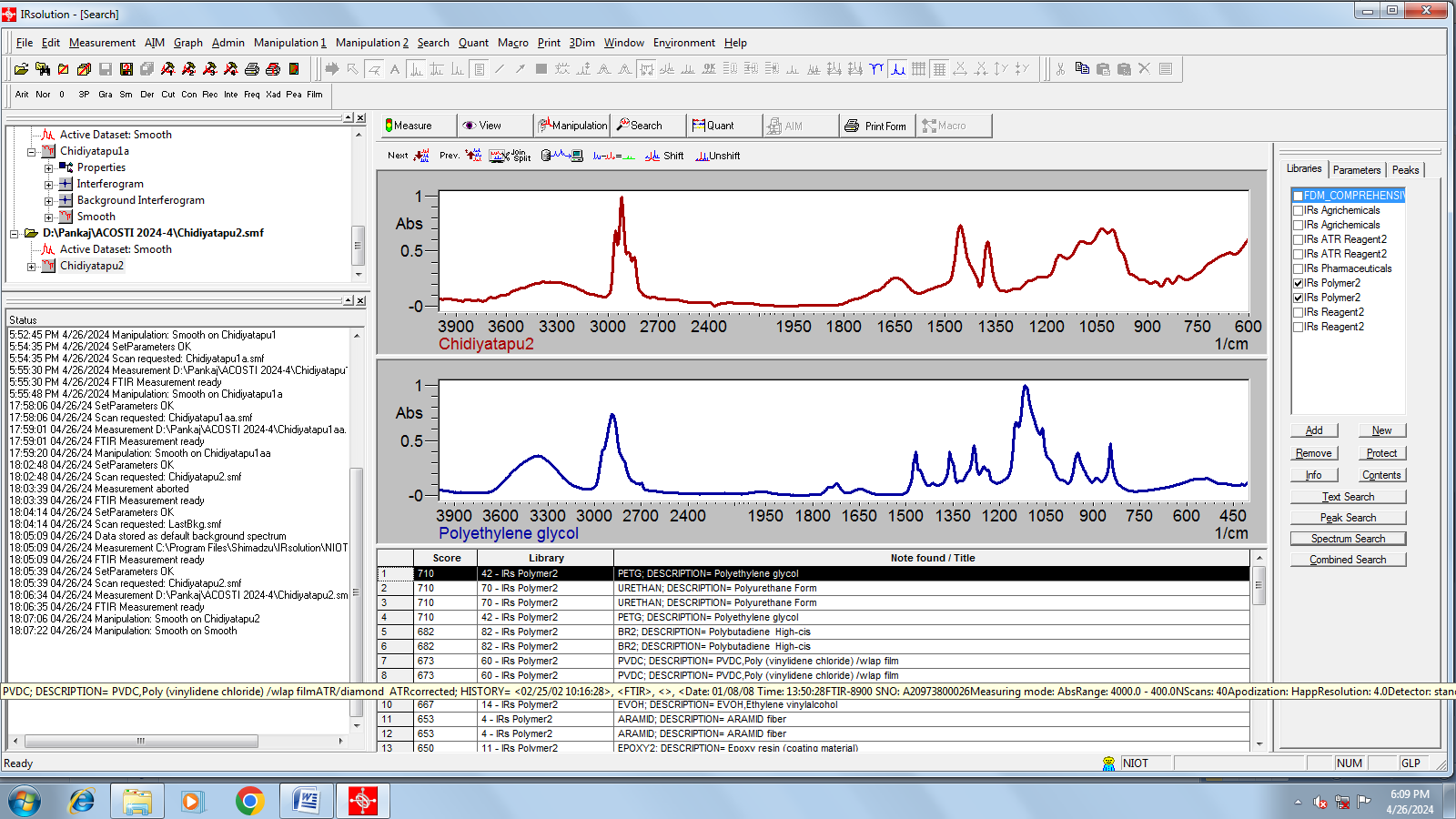 |
| **3** | Chidiyatapu 2a | ARAMID fiber | 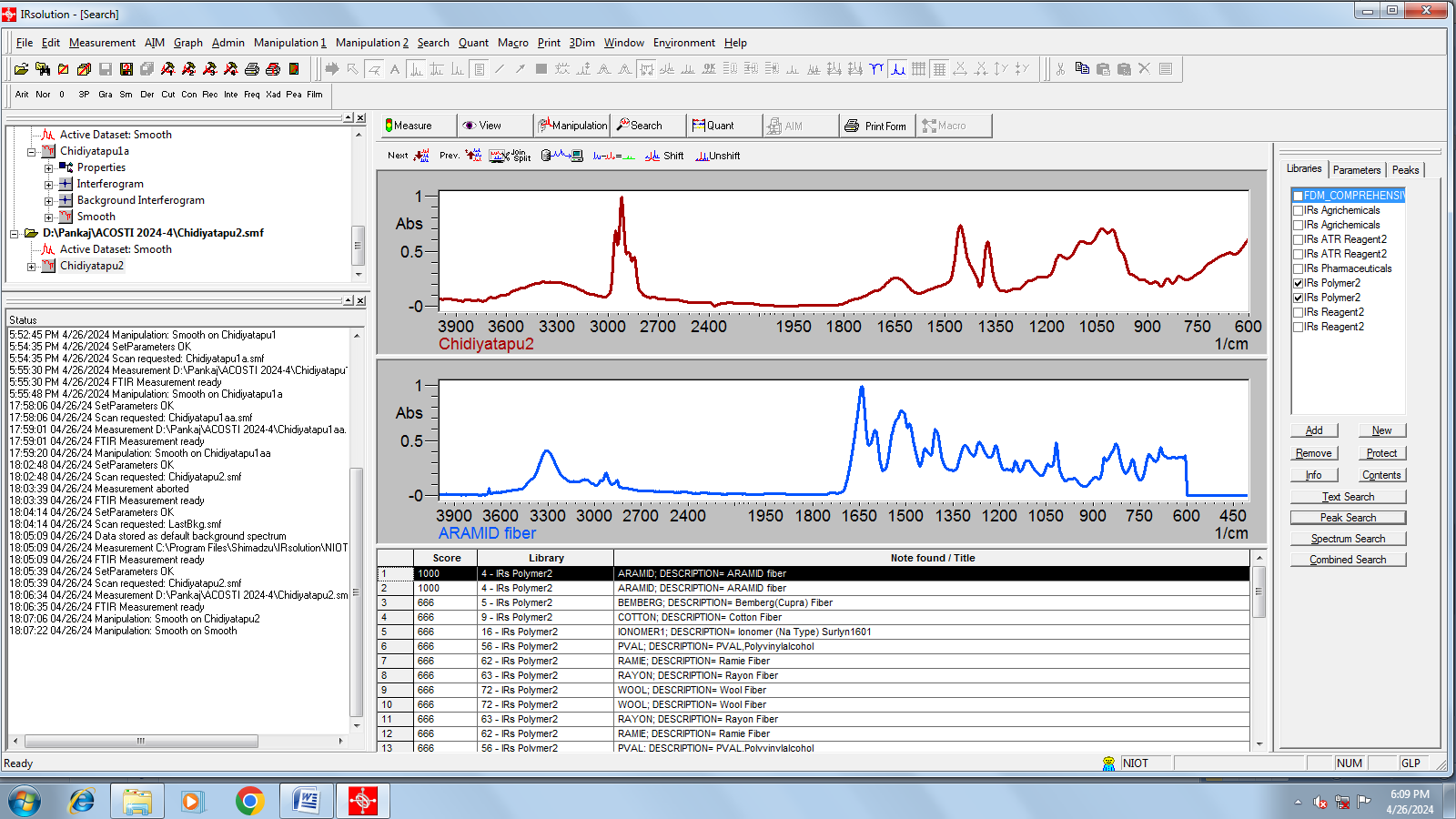 |
| **4** | Chidiyatapu 3 | Polybutadiene High-cis | 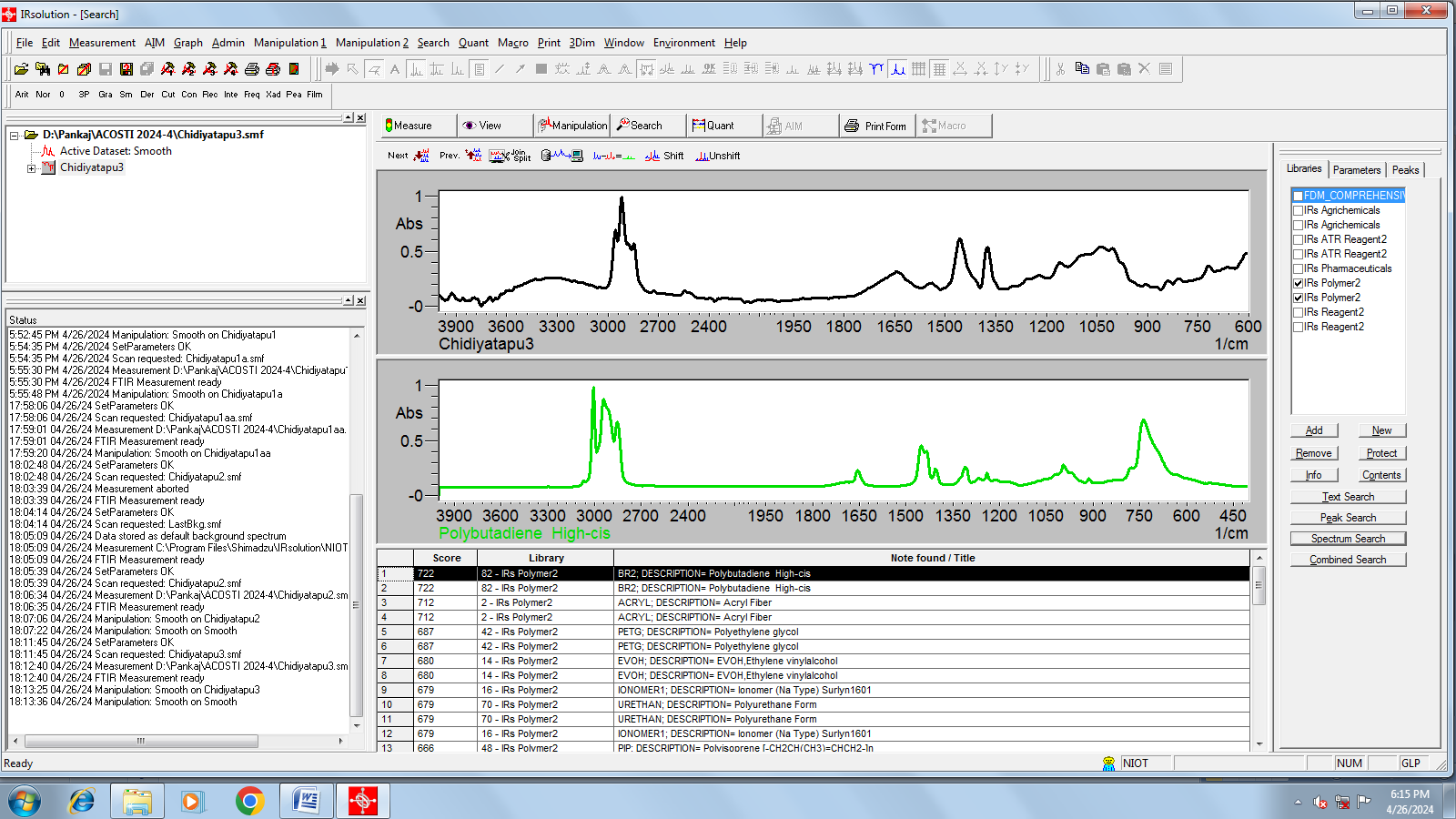 |
| **5** | Chidiyatapu 4 | ARAMID fiber | 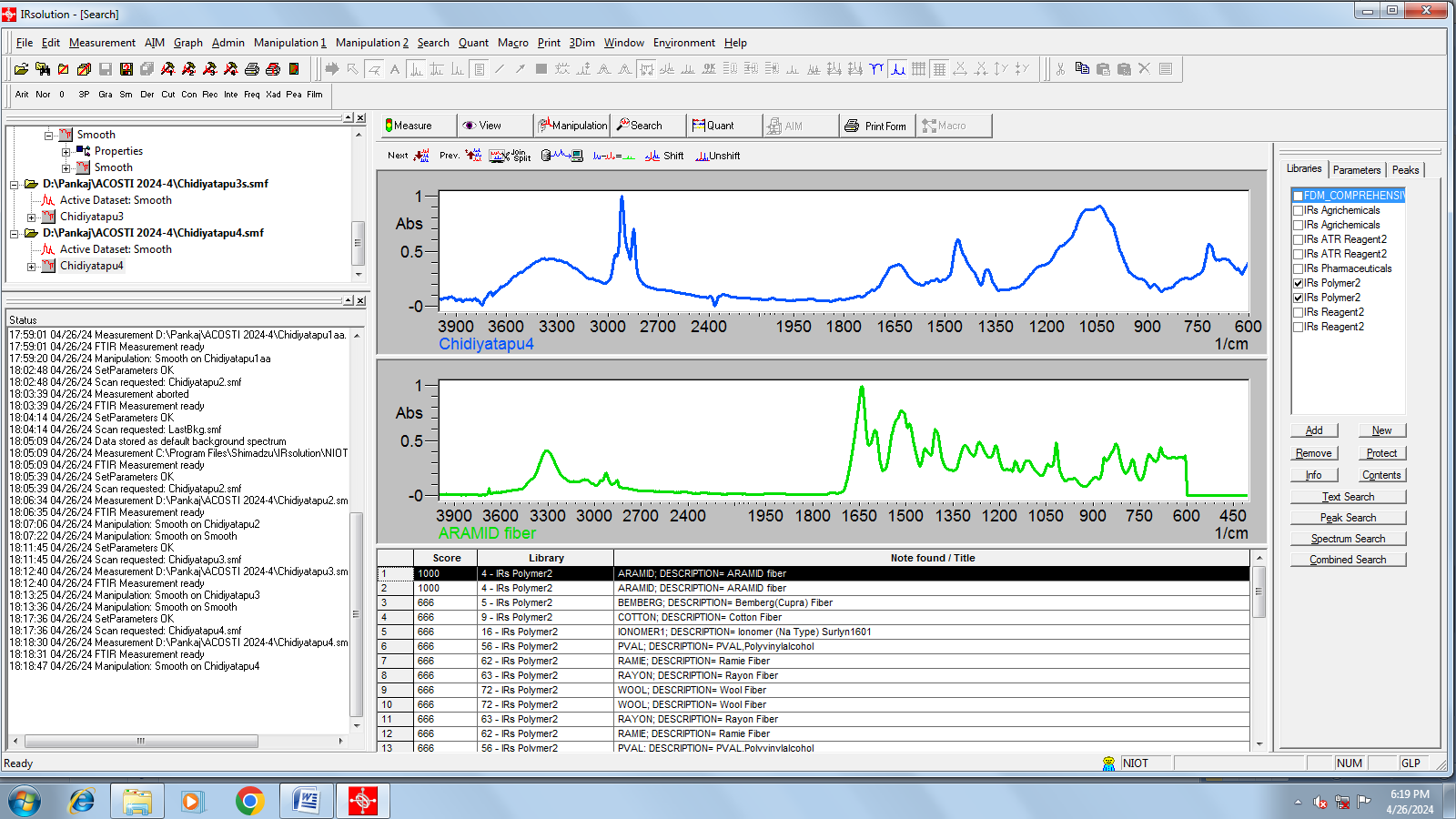 |
| **6** | Chidiyatapu 5 | Acrylonitrile – butadiene Rubber | 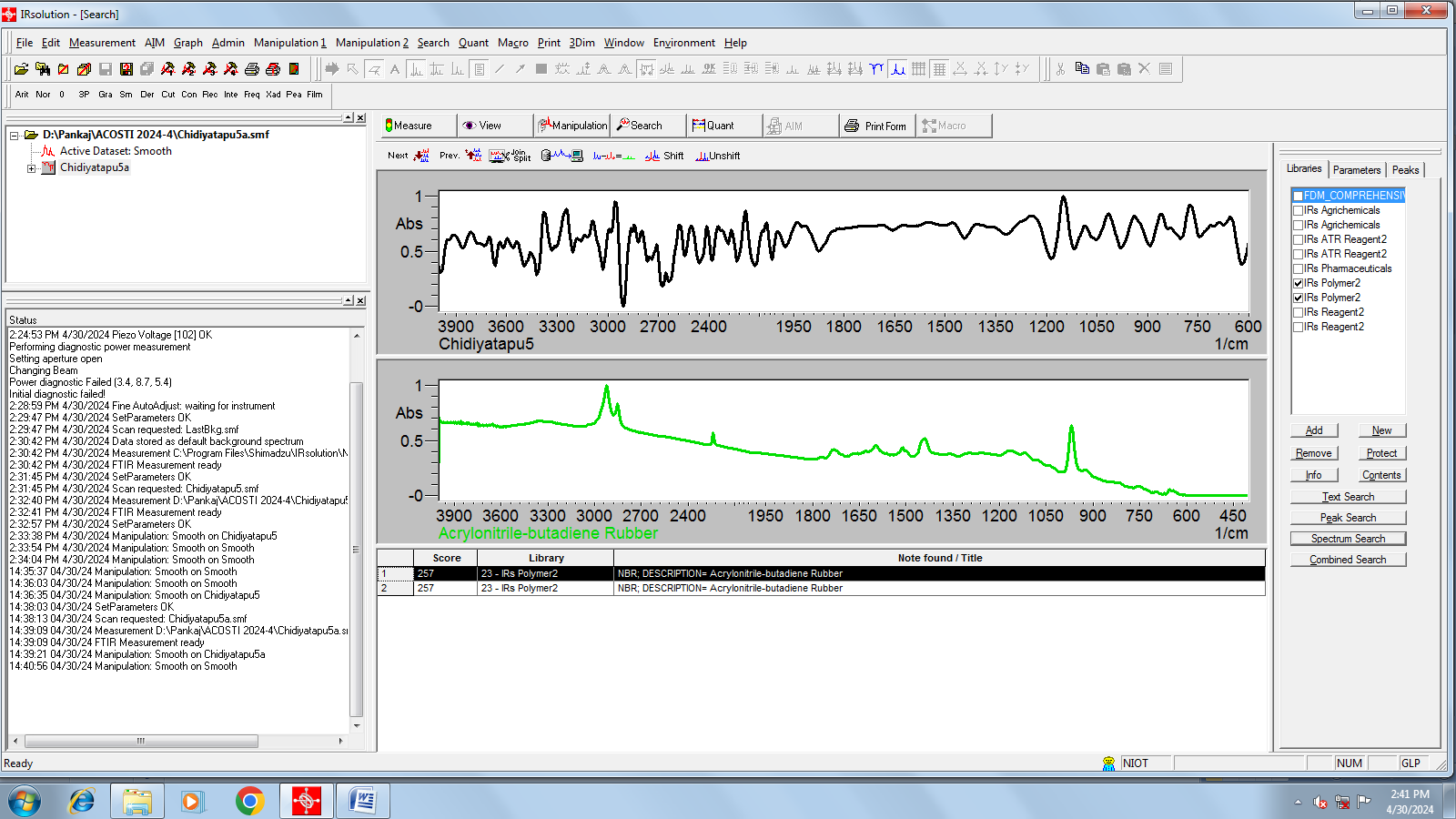 |
| **7** | Chidiyatapu 6a | ARAMID fiber | 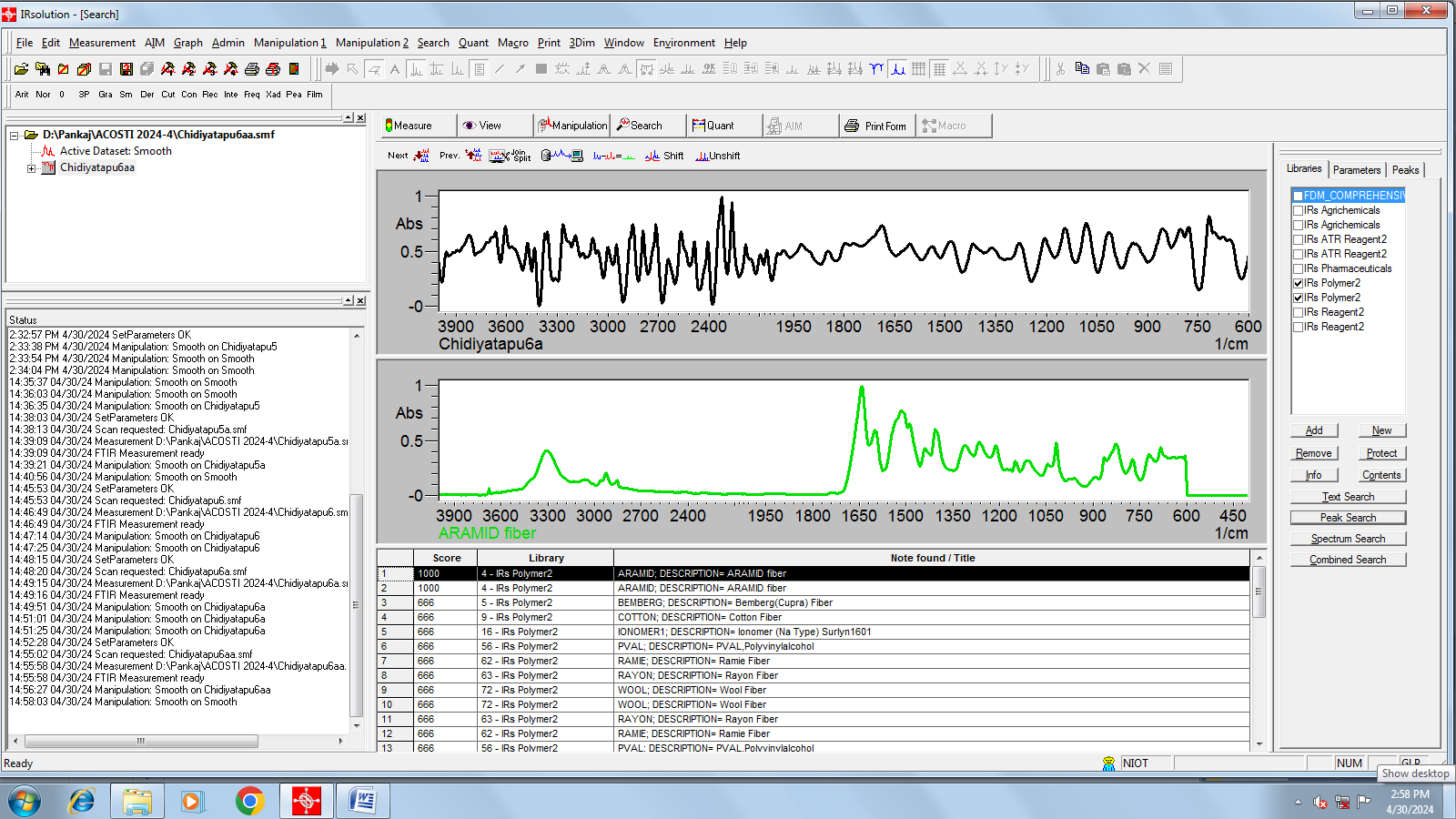 |
| **8** | Chidiyatapu 7 | Acrylonitrile – butadiene Rubber | 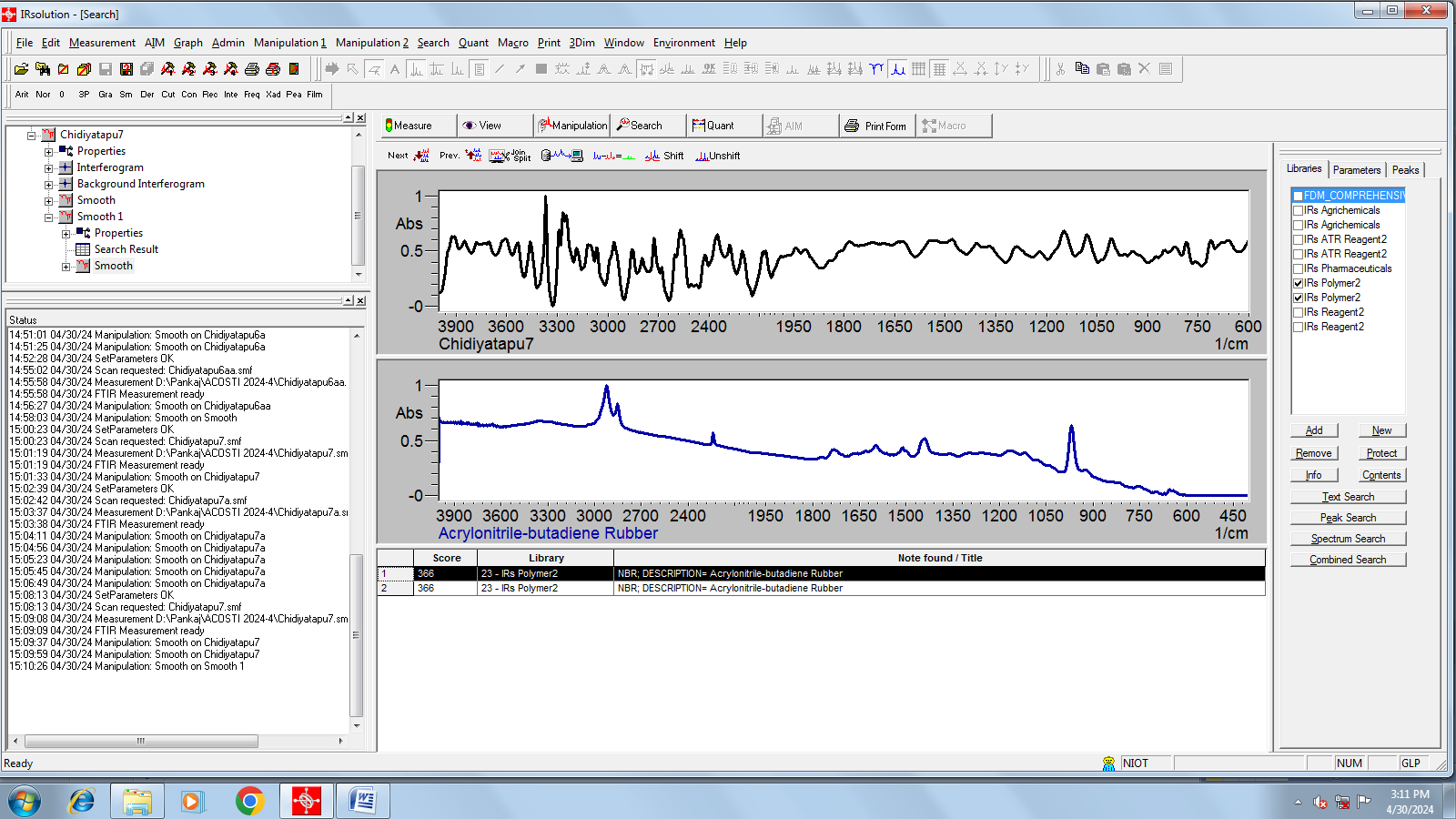 |

| **Location: Dignabad** | | | |
| --- | --- | --- | --- |
| **Sl. No** | **Sample ID and Information** | **Type of Microplastic** | **FTIR Result** |
| **1** | Dignabad 1 | Acrylonitrile – butadiene Rubber | 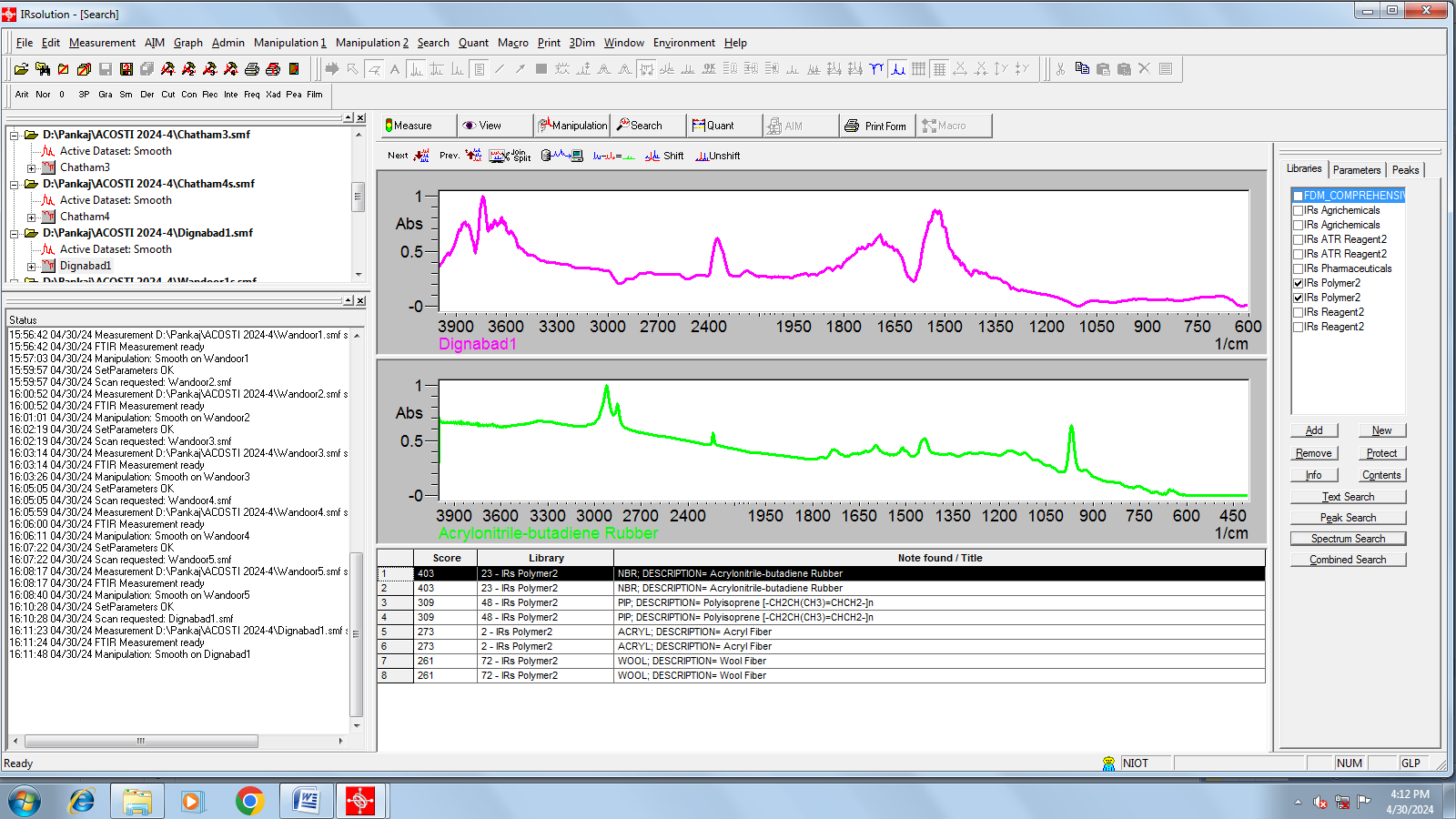 |
| **2** | Dignabad 2 | Polyisoprene [-CH2CH(CH3) =CHCH2-] n | 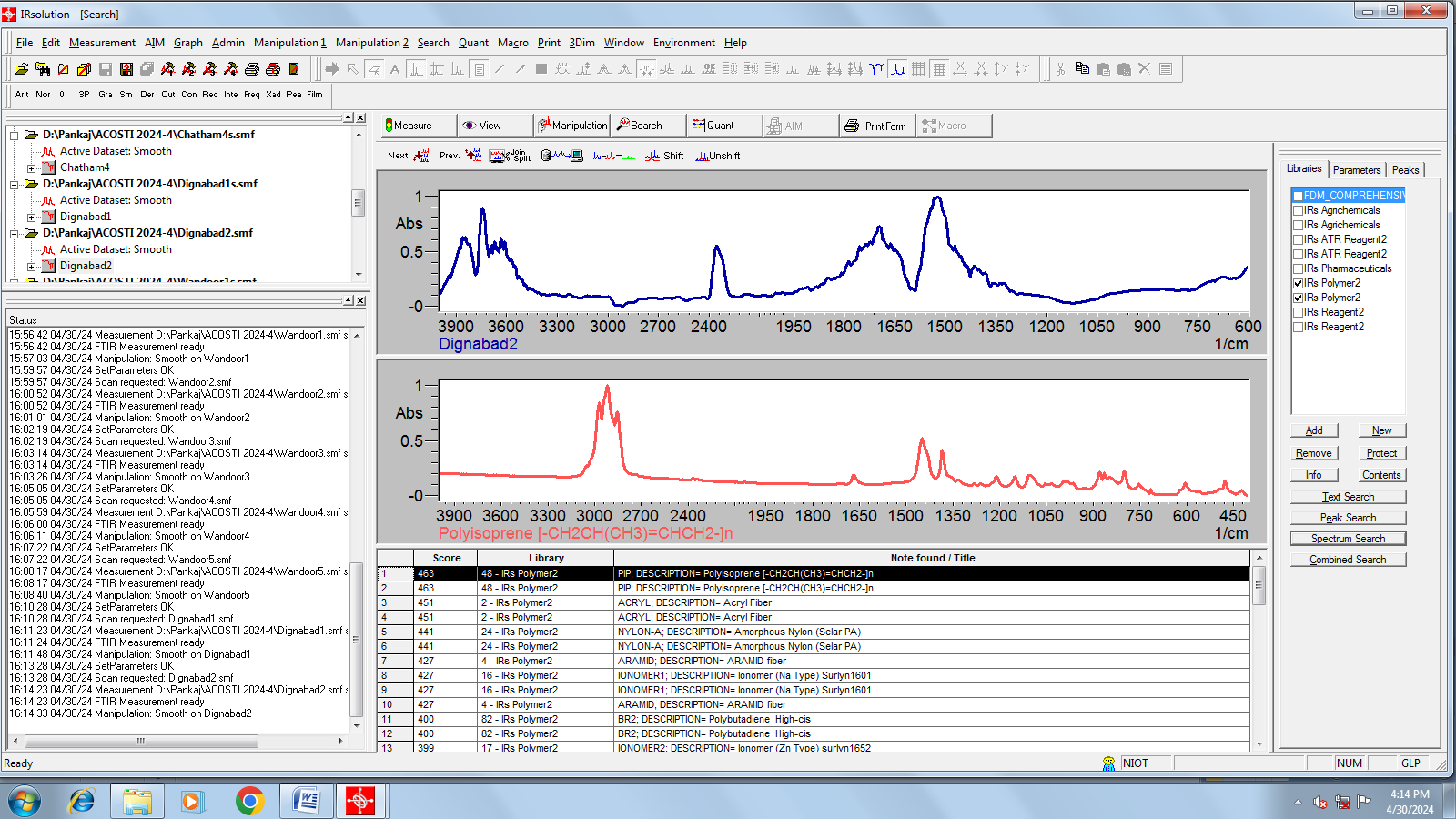 |
| **3** | Dignabad 3 | Pinene [C10H16] | 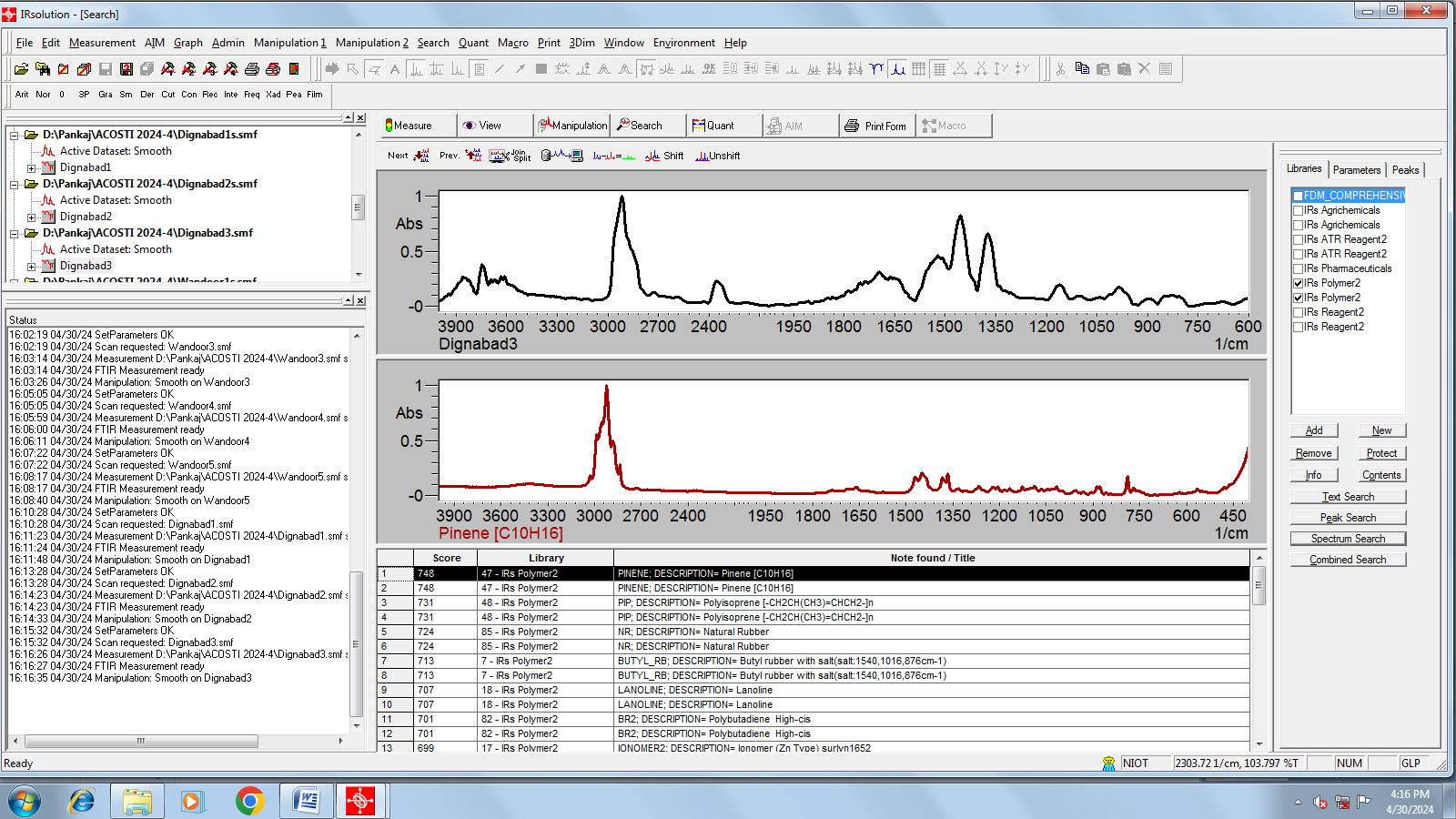 |
| **4** | Dignabad 4 | Polyisoprene [-CH2CH(CH3) =CHCH2-] n | 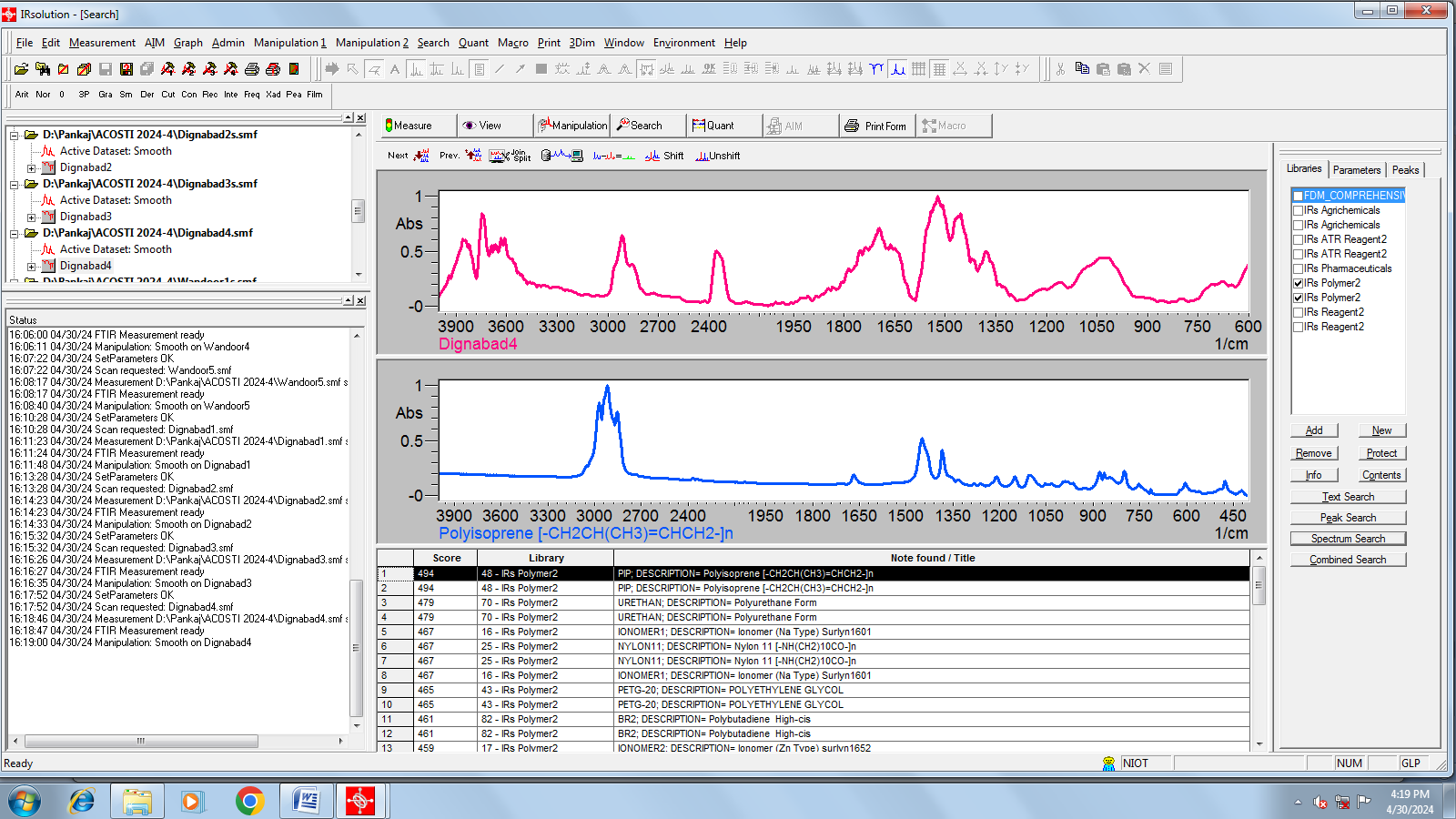 |
| **5** | Dignabad 5 | ARAMID fiber | 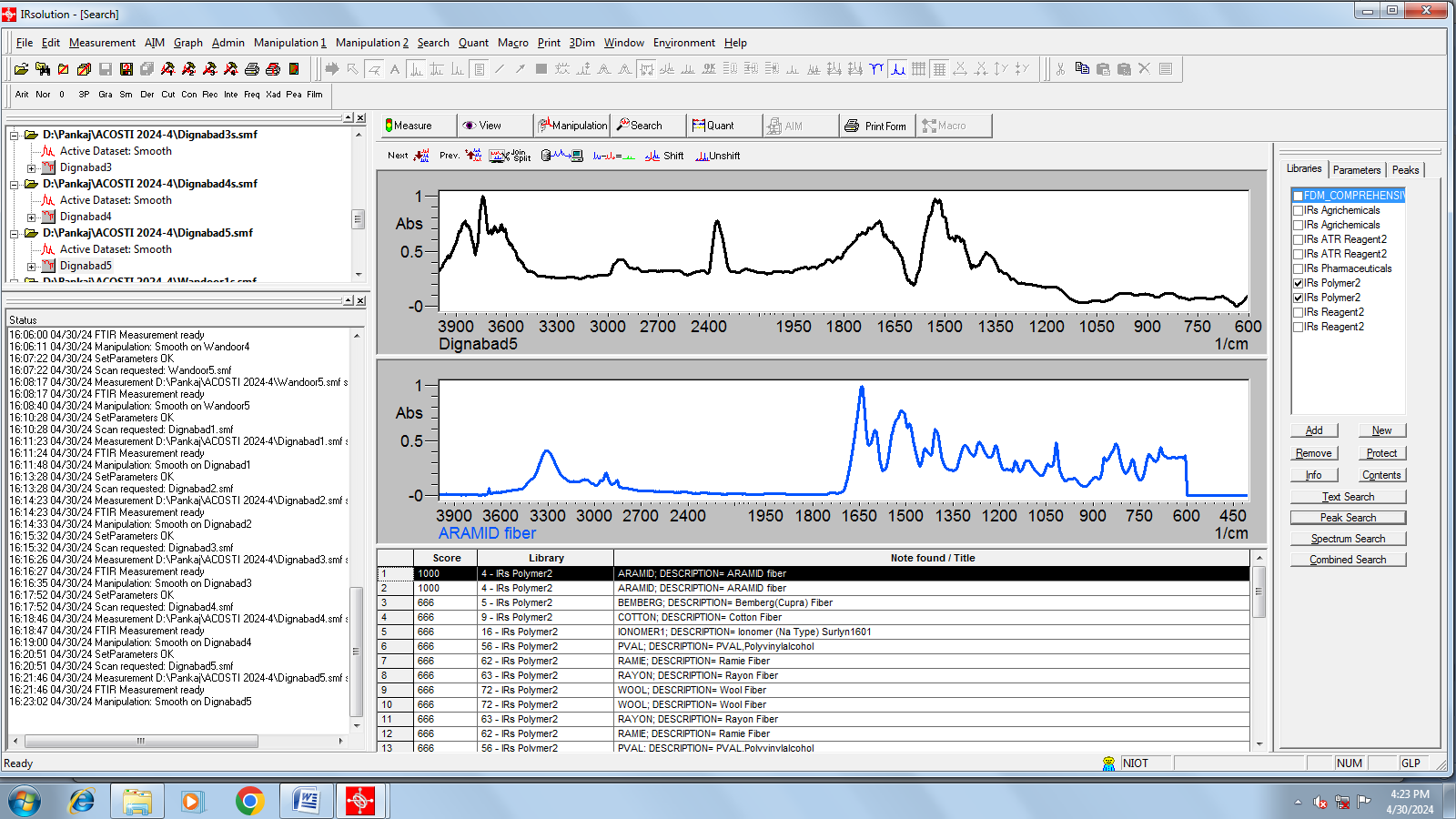 |
